# Hybrid Solar Spectral‐Splitting Photovoltaic‐Thermal Hydrogen Production Systems

**DOI:** 10.1002/advs.202503205

**Published:** 2025-04-27

**Authors:** Yu Tian, Pooria Hadikhani, Nada Alati, Bryce S. Richards, Gan Huang

**Affiliations:** ^1^ Institute of Microstructure Technology Karlsruhe Institute of Technology Hermann‐von‐Helmholtz‐Platz 1 76344 Eggenstein‐Leopoldshafen Germany; ^2^ Institute for Applied Materials – Electrochemical Technologies Karlsruhe Institute of Technology Adenauerring 20b 76131 Karlsruhe Germany; ^3^ Light Technology Institute Karlsruhe Institute of Technology Engesserstrasse 13 76131 Karlsruhe Germany

**Keywords:** concentrated photovoltaics, energy conversion, membrane‐less electrolyzer, photovoltaic‐thermal system, solar hydrogen, spectral splitting

## Abstract

Utilizing solar energy to produce green hydrogen is sustainable, but achieving high efficiencies remains challenging. In this study, a hybrid solar spectral‐splitting photovoltaic‐thermal hydrogen (SSPVTH) system is developed. Leveraging emerging membrane‐less electrolyzers, this system simultaneously employs photovoltaics and solar thermal energy to maximize solar‐to‐hydrogen (STH) production efficiency. The SSPVTH system based on gallium arsenide solar cells achieves an STH efficiency of 21.1%, representing a 31.1% relative improvement over a conventional PV‐electrolyzer that relies solely on photovoltaic electricity for water electrolysis. When equipped with perovskite photovoltaics, the system attains an STH efficiency of up to 19.0%. Additionally, with the integration of direct current power converters, the system maintains relatively stable performance across varying solar irradiance levels. Overall, this study provides a new design with the potential for achieving high‐efficiency hydrogen production through hybrid solar technologies.

## Graphical abstract

A hybrid solar photovoltaic‐thermal hydrogen system with membrane‐less electrolysis significantly boosts solar‐to‐hydrogen efficiency by co‐using thermal and electrical energy. The system attains good performance under variable irradiance with direct current power converters. When integrated with gallium arsenide or perovskite photovoltaics, it outperforms standard photovoltaic‐electrolyzer approaches, offering high‐efficiency hydrogen production.

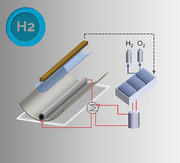

## 1 Introduction

Hydrogen presents a vision of zero‐pollution, sustainable, low‐cost, and high‐efficiency energy production.^[^ ^1^ ^]^ making it pivotal in addressing climate change and achieving global decarbonization goals. It has high potential for hard‐to‐decarbonize sectors such as transportation, industry, and energy storage, where it can replace fossil fuels, reduce greenhouse gas emissions, and provide long‐term energy solutions.^[^ ^2^, ^3^, ^4^ ^]^ Despite these promising attributes, the reality of hydrogen production processes today often contradicts this ideal, falling short of being low‐carbon and environmentally friendly. As of the end of 2022, a significant portion of global hydrogen production is derived from fossil fuels: nearly 62% from natural gas and 21% from coal – both without carbon capture, utilization, and storage – and 16% from by‐product hydrogen in refining and petrochemical industries.^[^ ^5^ ^]^ These methods produce what is known as ‘black’ and ‘gray’ hydrogen,^[^ ^6^ ^]^ respectively, neither of which aligns with the sustainable and green credentials that hydrogen energy promises to deliver. Hydrogen production from renewables remains relatively small, accounting for less than 1%.^[^ ^5^ ^]^

To solve this issue, a variety of clean energy sources are being explored for hydrogen production, with the combination of renewable electricity and water electrolysis emerging as a particularly promising approach.^[^ ^7^ ^]^ This strategy leverages the conversion of water into hydrogen and oxygen through the application of electrical energy that is derived from clean resources, ensuring that the hydrogen production process is free from carbon emissions and environmentally harmful by‐products. Among the diverse types of clean electricity being harnessed for hydrogen production, wind power and photovoltaics (PV) have been at the forefront of research and implementation efforts. Due to decades of continuous innovation and optimization, and thanks to the economies‐of‐scale of PV module production, conversion efficiencies have significantly improved,^[^ ^8^ ^]^ costs have been progressively lowered,^[^ ^9^ ^]^ and lifespans have been extended.^[^ ^10^ ^]^ These advancements make PV an increasingly attractive option for sustainable energy production. On the other hand, the deployment of PV systems offers remarkable modularity, and they can be installed in various settings, ranging from small‐scale residential homes to extensive solar farms, making electricity generation adaptable to different scales and contexts.^[^ ^11^ ^]^ This adaptability enhances the flexibility of deploying hydrogen production systems based on PV‐electrolyzer technology. As a further advancement, combined photovoltaic‐thermal (PVT) technology enables the simultaneous harnessing of solar energy for both electricity and heat production.^[^ ^12^ ^]^

Two factors influencing the solar‐to‐hydrogen (STH) conversion efficiency are the effectiveness of solar cells in transforming solar energy into electricity, and the performance of electrolysis systems in converting that electricity into hydrogen.^[^ ^1^ ^]^ Most recent research has focused extensively on enhancing the efficiency of both components. For solar harvesting, technologies such as solar‐concentrated multi‐junction PV cells, which achieve efficiencies exceeding 30%, have been employed in solar hydrogen systems.^[^ ^13^, ^14^ ^]^ By directly connecting indium gallium phosphide/gallium arsenide/germanium (InGaP/GaAs/Ge) triple‐junction cells (31% electrical efficiency under 23 suns) to an electrolyzer, the STH efficiencies of 24.4% can be achieved.^[^ ^15^ ^]^ However, maintaining this efficiency becomes challenging when solar radiation fluctuates. Optimal performance can only be sustained when the voltage between solar cells and electrolyzers is properly matched.^[^ ^15^ ^]^ Further efforts are required to ensure proper matching between solar cells and electrolyzers across varying solar irradiance levels. The inevitable optical losses of the solar concentrator must also be taken into account when designing concentrating PV‐EC devices.^[^ ^1^, ^13^ ^]^ For water electrolysis systems, proton exchange membrane (PEM) electrolyzers with efficiencies between 65% and 82% are usually utilized.^[^ ^16^ ^]^ Some devices based on PEM electrolyzers, and multi‐junction solar cells have shown good hydrogen production performance, but the scale and power of most of them are still limited.^[^ ^14^ ^]^ A recent notable work successfully demonstrates a kW‐scale solar hydrogen production system.^[^ ^13^ ^]^ However, the authors indicate that more than half of the incident solar energy is still not fully utilized and is dissipated as waste heat or converted into low‐grade thermal energy, due to the limited spectral response of the solar cells.

To further improve STH efficiency and provide synergistic benefits, thermal management has been integrated into solar hydrogen systems in some studies.^[^ ^13^, ^17^, ^18^ ^]^ Specifically, thermal management facilitates PV cooling, thereby increasing electrical efficiency, while simultaneously providing additional heating to reactants and catalysts in electrolyzers. This approach lowers overpotentials during electrolysis and enhances the hydrogen production rate.^[^ ^19^ ^]^ However, integrating thermal management into solar hydrogen designs still faces significant challenges. First, the heat extracted from PV cells is typically limited to temperatures below 80 °C to avoid overheating issues, which restricts the potential for further improving STH efficiency by heating the electrolyte. Second, the operating temperature of PEM electrolyzers is also limited to ≈80 °C due to the thermal instability of their membranes.^[^ ^13^ ^]^ As a result, even when high‐temperature heat is available, conventional PEM electrolyzers cannot effectively utilize it. While higher‐temperature solar hydrogen generation is thermodynamically advantageous, addressing these challenges requires significant innovation.

In contrast to previous studies, this work uniquely integrates advanced spectral and thermal management strategies to overcome these limitations. Specifically, a novel concept of a hybrid solar hydrogen system is proposed, termed the solar spectral‐splitting photovoltaic‐thermal hydrogen (SSPVTH) system, to achieve higher STH efficiency. Unlike conventional PVT systems, the SSPVT system separates the solar spectrum into distinct parts, directing high‐energy photons to PV cells for electricity generation and low‐energy photons to thermal absorbers for high‐temperature heat production.^[^ ^20^, ^21^, ^22^ ^]^ Moreover, the system incorporates recently developed membrane‐less electrolyzer barriers,^[^ ^23^, ^24^ ^]^ which operate at significantly higher temperatures by using fluid forces to separate electrolysis gas products instead of relying on solid membrane barriers. While these electrolyzers currently face challenges, such as higher ohmic losses, their compatibility with high‐temperature heat and electricity presents an exciting opportunity for next‐generation hybrid solar hydrogen systems. This innovative design addresses the previously underutilized thermal energy component in solar hydrogen systems and optimizes energy utilization across the solar spectrum.

To demonstrate this concept, single‐junction solar cells–based on both GaAs and perovskite absorbers – are employed in a concentrated solar system with a concentration ratio of 17. The SSPVTH system using GaAs PV can achieve an STH efficiency of 21.1%, which is 31.1 rel. % higher than a solar hydrogen system based on the same PV technology but without heat generation. More importantly, with the help of a direct current‐to‐direct current (DC‐to‐DC) converter, the system is able to stably maintain high STH efficiency under various solar irradiances, demonstrating the system's great potential for next‐generation high‐efficiency solar hydrogen production.

## 2 Results

### 2.1 Spectral‐Splitting Photovoltaic‐Thermal Hydrogen (SSPVTH) System Concepts

**Figure 1:**
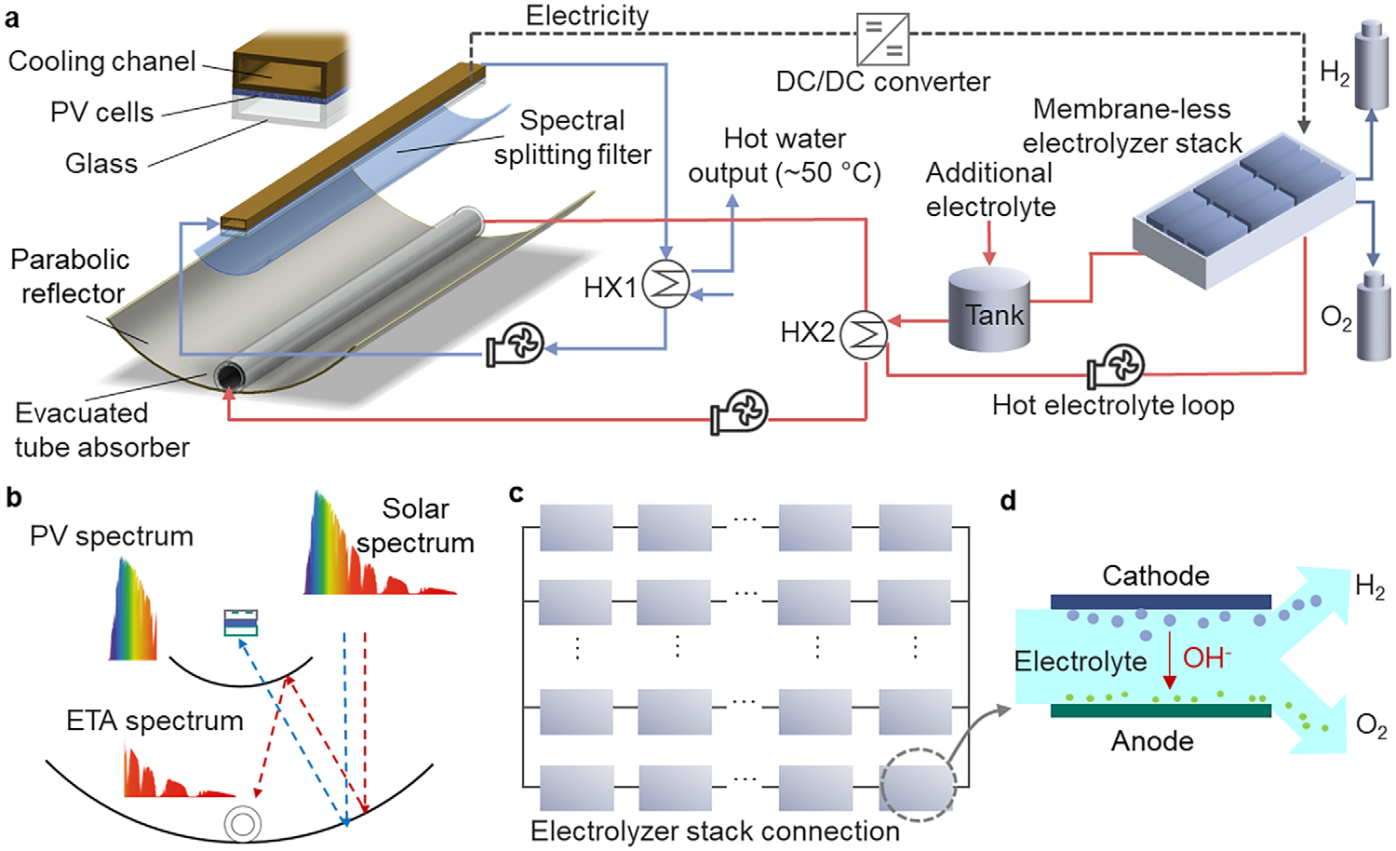
Schematic illustration of the spectral‐splitting photovoltaic‐thermal hydrogen (SSPVTH) production system. a) Concept of SSPVTH system integrated the parabolic reflector, spectral‐splitting filter, photovoltaics (PV), solar evacuated tube absorber (ETA), and membrane‐less electrolyzer stack. The SSPVTH system employs concentration and spectral splitting to generate high‐temperature heat from irradiance at wavelengths that cannot be utilized by PV cells. This high‐temperature heat is then used to heat the electrolyte in the electrolyzer, thereby enhancing the system's hydrogen production efficiency. b) Spectral‐splitting process for PV and ETA. The spectral‐splitting filter divides the solar spectrum into two parts. One part is directed to PV modules to generate electricity and the PV waste heat is used for domestic hot water, while the remaining portion, which cannot be utilized by PV, is directed to the ETA to generate heat. c) The electrical series / parallel connection of membrane‐less electrolyzer cells inside the stack. d) Working principle of the membrane‐less electrolyzer cell. The electrolyzer uses fluidic forces, rather than solid barriers, to separate the gas products of electrolysis.

A hybrid spectral‐splitting photovoltaic‐thermal hydrogen (SSPVTH) system is designed, whose main features include PV cells, solar thermal absorbers, spectral‐splitting filters, and membrane‐less electrolyzers, as shown in **Figure** **1**a. A solar cell measuring 50 × 80 mm was selected, with 30 cells connected in series and two strings in parallel to form a 2.4 × 0.1 m PV module. The design first collects sunlight through a parabolic reflector with dimensions of 2.4 × 1.8 m, with the dimensions chosen based on the length of the PV module and target concentration ratio. The sunlight reflects and concentrates it onto a spectral‐splitting optical filter. Above the optical filter is the PV module with integrated cooling channels, which have an area equal to that of the PV module. These cooling channels allow a heat transfer fluid (HTF) to remove waste heat from the PV cells, which can be used to produce domestic hot water at 50 °C via a heat exchanger (HX), illustrated as HX1 in Figure 1a.

Mounted to the parabolic reflector is an evacuated tube absorber (ETA) that generates high‐temperature heat, consisting of an outer glass tube with a diameter of 4.2 cm and an inner solar thermal absorber tube with a diameter of 3.2 cm. The HTF flows through the ETA and transfers the high‐temperature heat to the electrolyte via the HX2. The heated electrolyte then enters the membrane‐less electrolyzer stack, which consists of several electrolyzer cells connected in series and parallel based on the I‐V curve of PV modules, and using the electricity generated by the PV cells and high‐temperature heat generated by the ETA, produces hydrogen. The outlet temperature of HX2 is designed to exceed 180 °C, using thermal oil as the heat transfer fluid. This temperature range is ideal for hydrogen production in our system, as operating the membrane‐less electrolyzer at such elevated temperatures significantly enhances its performance. In contrast, the HX1 outlet temperature is ≈50 °C, which is more suitable for domestic hot water applications rather than hydrogen generation.

In order to co‐generate electricity and high‐temperature heat simultaneously, a spectral‐splitting optical design is essential, as illustrated in Figure 1b. The air mass 1.5 direct (AM 1.5D) solar spectrum (900 W m^−^^2^), first reaches a parabolic reflector with a concentration ratio of 17. The concentrator then reflects the light onto a parabolic spectral‐splitting optical filter with an appropriate cut‐off wavelength *λ*_cut_. The solar spectrum with wavelengths shorter than *λ*_cut_ passes through the optical filter to the PV cells, generating electricity along with waste heat for producing hot water. The solar spectrum with wavelengths longer than *λ*_cut_ is reflected by the optical filter to the ETA, generating high‐temperature heat. This heat is then used to heat the electrolyte in the electrolyzer.

The current‐voltage (I‐V) performance of the electrolyzer stack can be determined by superimposing the series and parallel circuits of the cells to generate the I‐V curve of the entire stack. The relationship between the series and parallel connections is depicted in Figure 1c. Several cells are connected in series to form a unit, with the number of series connections controlling the voltage of the entire stack. Multiple units are then connected in parallel to form the stack, with the number of parallel units determining the current value of the entire stack. In a directly connected PV‐electrolyzer hydrogen production system, the operating point (OP) is the intersection of the I‐V curve of the PV module and the I‐V curve of the electrolyzer. To achieve optimal hydrogen production efficiency, it is important to ensure that this OP is as close to the maximum power point (MPP) of the PV module as possible. Therefore, for a given PV module, selecting the appropriate number of series‐connected cells in the electrolyzer unit and the number of parallel units in the stack to adjust the I‐V curve of the entire electrolyzer stack is crucial, which is detailed in the below sections.

The working principle of a membrane‐less electrolyzer cell is illustrated in Figure 1d. The electrolyzer relies on fluidic flow for hydrogen and oxygen separation. Hydrogen and oxygen are produced on the cathode and anode, respectively, and collected on the corresponding electrode sides. In this study, each cell had an electrode area of 5 cm^2^, and the electrolyte used was a 30% (w/w) potassium hydroxide (KOH) solution.

### 2.2 Light/Thermal Management and Performance of the SSPVTH System with GaAs PV

**Figure 2:**
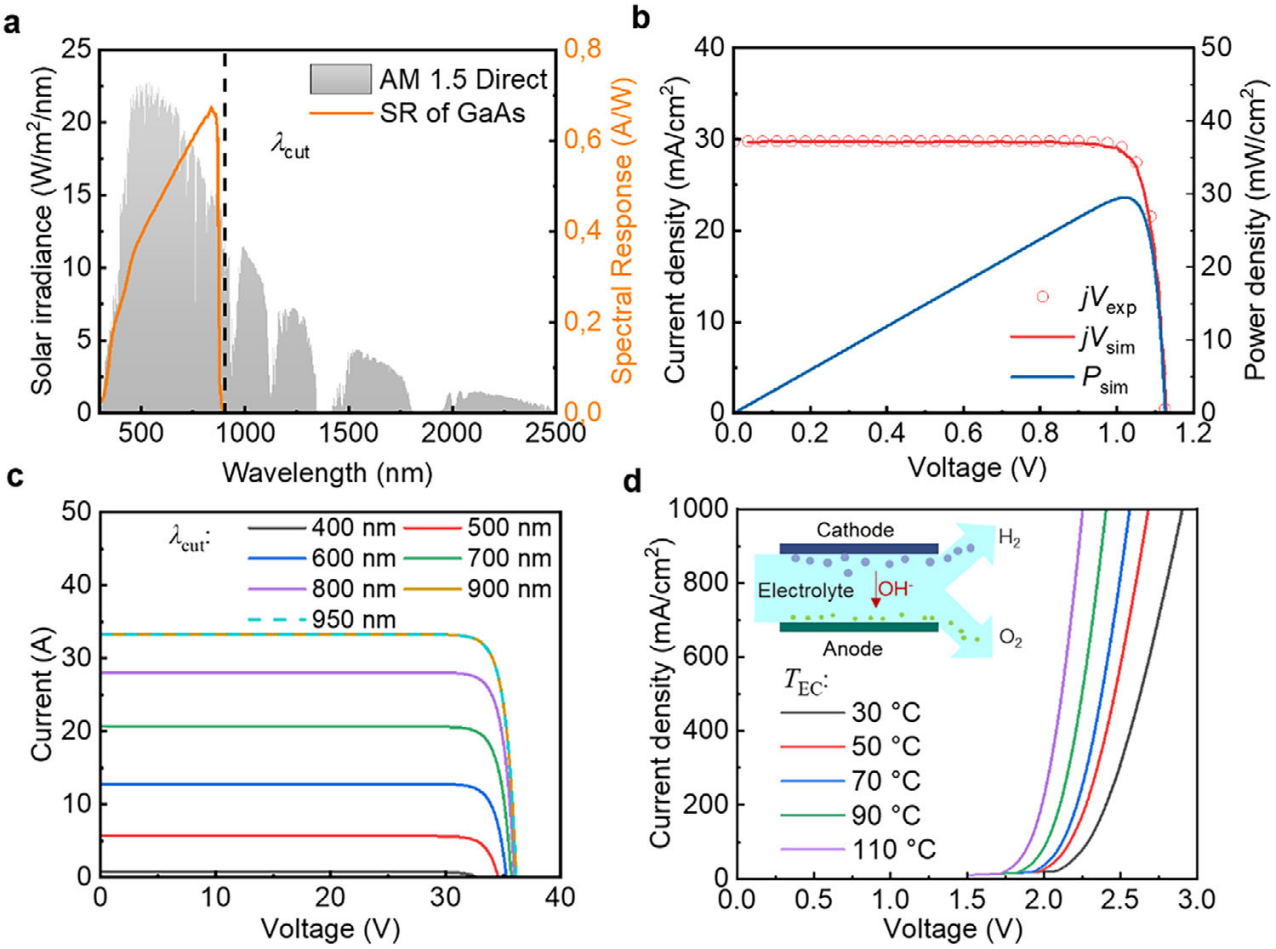
Light and thermal management in the SSPVTH system with the GaAs PV. a) The relationship between air mass 1.5 direct (AM 1.5D) solar spectrum, the spectral response of GaAs PV, and the cut‐off edge (*λ*_cut_) of the spectral‐splitting filter. b) Comparison of simulated current density‐voltage (jV) curves of the PV with reported experimental data. The simulation results show that the efficiency of GaAs PV is 29.5%, which agrees well with the efficiency reported in the literature, which is 29.1%. c) The impact of *λ*_cut_ on the I‐V curve of GaAs PV. The I‐V curve no longer changes after *λ*_cut_ exceeding 900 nm. d) The effect of electrolyte temperature on the I‐V curve of the electrolyzer. As the temperature increases, the voltage required at the same current density (same hydrogen production rate) decreases, indicating an increase in efficiency.

Record‐efficiency GaAs single‐junction solar cells were initially selected for use in the modeled SSPVTH system. GaAs solar cells are selected for the SSPVTH system due to their high efficiency and robust performance under concentrated sunlight, making them ideal candidates for high‐performance concentrated solar hydrogen conversion systems. GaAs solar cells have an energy bandgap (*E*_g_) of ≈1.4 eV (≈900 nm). Consequently, a substantial portion of the infrared solar spectrum (> ≈900 nm), which cannot be utilized for electricity generation, can be effectively directed toward the ETA to generate high‐temperature heat. This approach enhances the overall energy utilization of the system. The relationship between AM1.5D, the spectral response of GaAs PV, and the cut‐off edge (λ_cut_) of the spectral‐splitting filter is shown in **Figure** **2**a. Ensuring that the solar irradiance generates sufficient power is the primary goal. Therefore, the cut‐off edge is selected at 900 nm to minimize the effect of spectral splitting on electricity generation. The filter is assumed to behave as a near‐ideal short pass, with an estimated 5% light absorption. Specifically, the transmittance before the cut‐off edge is 95%, while the reflectance after the cut‐off edge reaches 95%. The standard PV diode model is used to simulate the current density‐voltage (j‐V) curve of a PV cell, as detailed in the Method section. The simulation results in Figure 2b, based on AM1.5D conditions and a temperature of 25 °C, exhibit an efficiency of 29.5%, which agrees well with the reported experimental data of 29.1%.^[^ ^25^ ^]^

To verify that 900 nm is the appropriate cut‐off edge, the I‐V curves of the PV modules under different cut‐off edges were simulated, as shown in Figure 2c. The I‐V curves of a module are based on the jV curve of each cell, obtained through the series‐parallel relationship according to the geometry area and connection of the PV modules in the system. This result is consistent with the known influence of light intensity changes on the I‐V performance of PV modules.^[^ ^26^, ^27^ ^]^ When the cut‐off edge reaches 900 nm, the PV module achieves optimal I‐V performance. Beyond this point, further increases in the cut‐off edge wavelength do not alter the I‐V curve but will reduce the solar energy for ETA high‐temperature heat generation. Therefore, the cut‐off edge of 900 nm is selected in this research.

In order to analyze the STH efficiency of the system, it is also important to know the effect of temperature on the performance curve of the electrolyzer and PV. The effect of temperature on the electrolyzer performance is shown in Figure 2d, and the PV efficiency as a function of temperature is detailed in the Method section. As the temperature rises, the voltage required at a given current density (which corresponds to a constant hydrogen production rate) decreases. This decrease in required voltage translates to lower electrical power consumption, thereby making higher temperatures beneficial for the electrolysis process. In contrast to electrolyzers, high temperatures reduce the efficiency of PV cells. Different PV technologies exhibit varying levels of sensitivity to high temperatures. For instance, the temperature coefficient of crystalline silicon PV is −0.29%/°C,^[^ ^28^ ^]^ while some PV technologies perform better under high temperatures. GaAs PV, for example, has a temperature coefficient as low as −0.08%/°C.^[^ ^28^ ^]^

**Figure 3:**
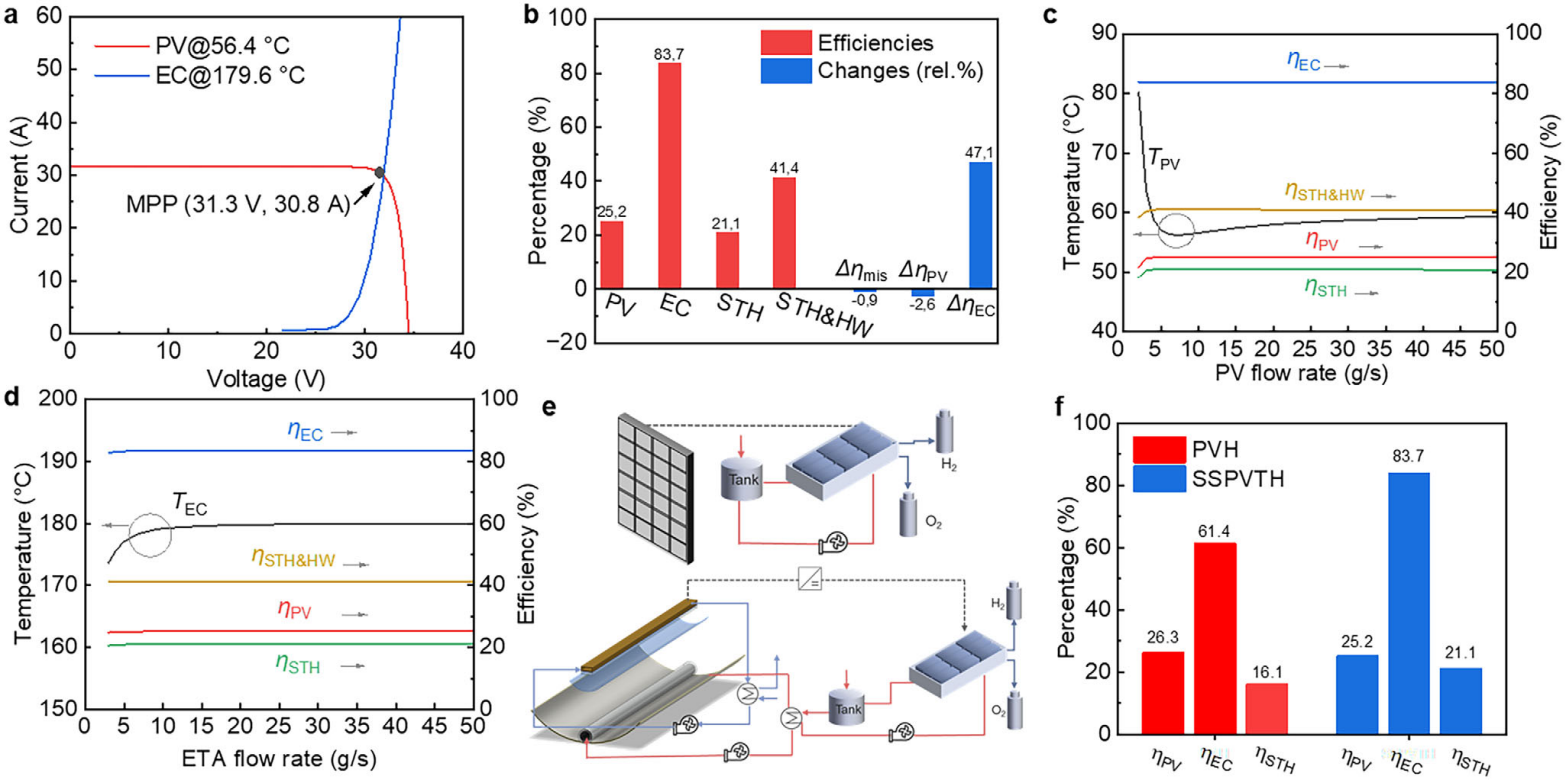
Performance of the SSPVTH system with the GaAs PV. a) Current‐voltage curves of PV and electrolyzer under optimal conditions. Under this condition, the temperature of the PV is 56.4 °C, and the temperature of the electrolyzer is 179.6 °C. b) The efficiencies of PV, electrolyzer (EC), STH, and STH & hot water (STH&HW) in the system under optimal conditions. The STH efficiency achieves 21.1%. The reduction in STH efficiency (Δ*η*_mis_) due to the mismatch between the I‐V curves of the PV and the electrolyzer is only 0.9%. The decrease in PV efficiency (Δ*η*_PV_) resulting from overheating, compared to the standard temperature of 25 °C, is only 2.6%. Conversely, the improvement in electrolyzer efficiency (Δ*η*_EC_) due to heating the electrolyte is 47.1%. c) Effect of HTF flow rate in PV modules on PV temperature and system performance. d) Effect of HTF flow rate in ETA on electrolyzer temperature and system performance. Due to thermal decoupling, the flow rates in the PV and ETA can be controlled independently, allowing for adjustments to the temperatures of the PV and electrolyzer. e) Diagram of a conventional photovoltaic‐hydrogen (PVH) system and the SSPVTH system. f) Comparison of PVH and SSPVTH Performance. Both systems use record‐efficiency GaAs PV.

After determining the optimal cut‐off value and assessing the impact of temperature on the electrolyzer performance, the solar hydrogen production performance results for the GaAs PV‐based SSPVTH system can be obtained. **Figure** **3**a shows the I‐V curves of the PV module and electrolyzer stack under the configured condition, where 16 electrolyzer cells are connected in series as a unit, and 11 units are connected in parallel. The operating point is the intersection of the two curves. Even in a design with a concentration ratio of 17, the temperature of the PV can be maintained at only ≈56 °C, thanks to the HTF in the cooling channel behind the PV module. This low operating temperature does not significantly reduce the performance of the PV, with only a 2.6% relative drop in efficiency. Additionally, the low‐temperature coefficient of GaAs PV means that it is only slightly affected by the heating. The MPP of the PV module is also marked in the figure (i.e., 31.3V, 30.8 A). Meanwhile, the electrolyzer can reach a high temperature of 179.6 °C, which reduces the required voltage and shifts the I‐V curve to the left, leading to improved efficiency. Since the number of series and parallel connections in the electrolyzer stack has been optimized and is well matched to the PV under the ideal conditions, the OP and MPP are very close, thereby minimizing the STH efficiency loss caused by the mismatch between the OP and MPP.

More results are detailed in Figure 3b, where the red bars indicate the efficiency of each component and the overall system. This includes the efficiency of the PV and electrolyzer, the STH efficiency, and the overall efficiency including STH and hot water (STH&HW) production. Due to the optical loss of light before reaching the PV and the higher operating temperature compared to the standard temperature, the efficiency of the PV decreases to 25.2%. However, the efficiency of the electrolyzer reaches 83.7% at its operating temperature of 179.6 °C. The combination of these factors results in an overall STH efficiency of 21.1% for the system. For the hydrogen generated from the GaAs‐based SSPVTH system, the contribution of electricity is 81.9%, while the contribution from heat is 18.1% (Figure S1, Supporting Information). Additionally, considering the system's capability to produce by‐product hot water, the overall system efficiency is 41.4%. Additionally, the blue bars in Figure 3b illustrate the impact of mismatching and heating on the efficiencies of the STH, PV, and electrolyzer. The reduction in STH efficiency (Δ*η*_mis_) due to the mismatch between the I‐V curves of the PV and the electrolyzer (i.e., the mismatch between OP and the MPP) is only 0.9%. The decrease in PV efficiency (Δ*η*_PV_) resulting from overheating, compared to the standard temperature of 25 °C, is only 2.6%. Conversely, the improvement in electrolyzer efficiency (Δ*η*_EC_) due to heating the electrolyte from the standard temperature of 30 to 179.6 °C is 47.1%. The high STH efficiency is attributed to several factors: a well‐matching PV module and electrolyzer stack design, effective thermal management for cooling PV, and an increase in the electrolyzer temperature.

The HTF flow rates in PV and ETA play important roles. Figure 3c,d illustrates the optimization process of the HTF flow rate in both the PV and ETA. Because the system achieves thermal decoupling, the temperatures of the PV and the electrolyzer can be independently controlled by adjusting the HTF flow rates in the PV and the ETA, respectively. As the flow rate of HTF in the PV increases, the heat exchange process intensifies. The temperature of the PV drops rapidly from 80.2 °C at a flow rate of 2 g s^−1^ to 56.4 °C at a flow rate of 6 g s^−1^. At this point, the heat exchange is sufficient, and the temperature of the PV reaches its minimum value. Consequently, the efficiency of the PV, the STH efficiency, and the overall system efficiency reach their maximum, as shown in Figure 3c. As the flow rate continues to increase, the temperature of the PV rises slightly. This is because the HX1 is not efficient enough to transfer heat from the HTF to the constant 50 °C hot water output, resulting in a slight increase in the temperature of the HTF and the PV. Additionally, as the flow rate increases, the power consumed by the pump also increases, leading to a decrease in STH and system efficiency. Therefore, 6 g/s is determined to be the optimal flow rate of HTF in the PV.

Since the membrane‐less electrolyzer separates the produced hydrogen and oxygen through fluid mechanics, the electrolyte flow rate is a crucial variable that affects the gas crossover rate. Therefore, based on the results of the previous experimental work,^[^ ^24^ ^]^ the flow rate for each electrolyzer cell was set to 20 L/h (30.2 kg h^−1^), which corresponds to the scaled‐up value proportional to the electrode area used in the previous work, and then the total flow rate for each stack was calculated based on the number of units. With the electrolyte flow rate fixed, serving as the cold end in HX2, the HTF flow rate in the ETA is optimized as the only variable in this loop. Figure 3d indicates that as the flow rate of HTF in the ETA increases, the temperature of the electrolyzer gradually rises due to the increased heat transfer intensity in HX2. When the flow rate reaches 15 g/s, the temperature of the electrolyzer stabilizes and changes slightly. Therefore, 15 g/s is selected as the optimal flow rate of HTF in the ETA. At this point, the electrolyte temperature is at its highest at 179.6 °C, allowing the system to achieve a higher STH efficiency.

To better describe the impact of spectral and thermal management in this study, this system was compared to a conventional PV‐hydrogen (PVH) system, with the system designs shown in Figure 3e. In the PVH system that relies solely on PV electricity for water electrolysis, there is no parabolic reflector, spectral splitting filter, or ETA. For a fair comparison, the conventional PVH system also uses the same GaAs PV and electrolyzers as the SSPVTH system. The series and parallel connections of the PV cells and electrolyzer cells in the PVH system are optimized to minimize the mismatch loss (Δ*η*_mis_). The results of PVH are compared with those of SSPVTH in Figure 3f. Due to the absence of optical losses from a concentrator and optical filter in the PVH system, the PV cells in the PVH system exhibit higher efficiency compared to those in the SSPVTH system. However, the temperature of the electrolyzer is significantly lower than that in the SSPVTH system, at only 54 °C, resulting in a low efficiency of 61.4%, which is a considerable decrease compared to the electrolyzer in the SSPVTH system. Combining the influence of these two factors, the STH efficiency of the PVH system is 16.1%. The SSPVTH system achieves a 31.1% relative improvement in STH efficiency compared to the PVH system. This improvement is attributable to spectral and thermal management.

### 2.3 Performance of SSPVTH with Perovskite PV

**Figure 4:**
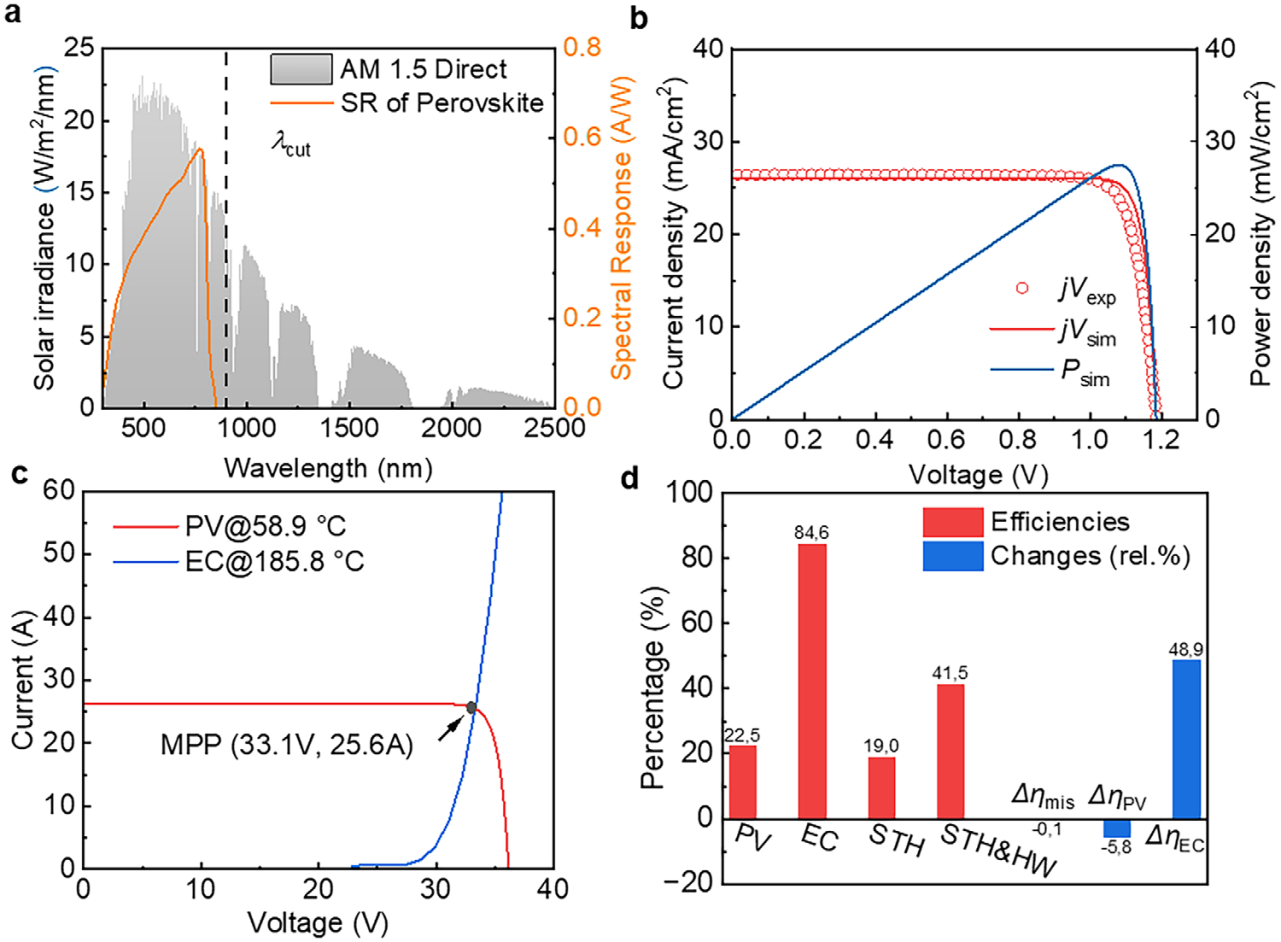
Results of SSPVTH system with perovskite PV. a) The relationship between Air Mass 1.5 direct, the spectral response of perovskite PV, and the *λ*_cut_ of the spectral‐splitting filter. b) Comparison of simulated performance curves of perovskite PV modules with reported experimental data. The simulation results show that the efficiency of perovskite PV is 25.2%, while the efficiency reported in the literature is 25.6%. c) I‐V curves of PV and electrolyzer under optimal conditions. The temperature of the PV is 58.9 °C, and the temperature of the electrolyzer is 185.8 °C. d) The efficiencies of PV, electrolyzer (EC), STH, and STH & hot water (STH&HW) in the system under optimal conditions. The STH efficiency achieves 19.0%. The reduction in STH efficiency (Δ*η*_mis_) due to the mismatch between the I‐V curves of the PV and the electrolyzer is only 0.1%. The decrease in PV efficiency (Δ*η*_PV_) resulting from overheating, compared to the standard temperature of 25 °C, is 5.8%. Conversely, the improvement in electrolyzer efficiency (Δ*η*_EC_) due to heating the electrolyte is 48.9%.

Perovskite PVs are also selected in this study as emerging photovoltaic materials to explore their potential in the SSPVTH system. The bandgap of perovskite PVs is tunable from 1.5 to 2.3 eV by adjusting the chemical composition, allowing for a considerable and adjustable amount of energy for solar thermal conversion. The bandgap of perovskite PV is tunable from 1.5 to 2.3 eV by varying the chemical components, ensuring a considerable and changeable amount of energy for solar thermal. The perovskite PV used in this study is based on one of the best‐performing compositions reported in recent research by Zhao et al.^[^ ^29^ ^]^ Specifically, a rubidium chloride (RbCl)‐doped formamidinium lead iodide (FAPbI_3_) perovskite is used. The selected perovskite PV has a bandgap of 1.53 eV. This perovskite PV demonstrated excellent long‐term performance, retaining 96% of its initial efficiency after 1000 h of shelf storage and 80% of its efficiency after 500 h of thermal aging at 85 °C. The fabrication of the perovskite PV follows a modified two‐step deposition method: first, a PbI_2_ seed layer is deposited, followed by the diffusion and annealing of a formamidinium iodide solution. Additionally, methylammonium chloride is introduced into the precursor to enhance crystallinity and film quality. The relationship between AM1.5D, the spectral response of perovskite PV, and the *λ*_cut_ of the spectral‐splitting filter is determined first, shown in **Figure** **4**a. The *λ*_cut_ here is also selected as 900 nm. After determining the *λ*_cut_, the jV and power simulation results of the perovskite PV are shown in Figure 4b. The simulated PV efficiency is 25.2%, while the actual efficiency reported in the literature is 25.6%.^[^ ^29^ ^]^

The I‐V curves of the PV and electrolyzer are shown in Figure 4c. The operating temperature of the PV is 58.9 °C and the operating temperature of the electrolyzer is 185.8 °C. Due to the lower efficiency of the perovskite compared to the previous GaAs, the perovskite‐based system achieved an STH efficiency of 19.0%. For the hydrogen generated from the perovskite‐based SSPVTH system, the contribution of electricity is 81.1%, while the contribution from heat is 18.9%. The efficiency of each component and the change in efficiency are shown in Figure 4d. The most significant impact is the decrease in PV efficiency, which is 22.5% for perovskite PV, lower than the efficiency of 25.2% for GaAs PV. The efficiency reduction caused by the mismatch between the I‐V curves of the PV and electrolyzer is only 0.1%.

### 2.4 Performance of SSPVTH Systems Under Different Irradiance

**Figure 5:**
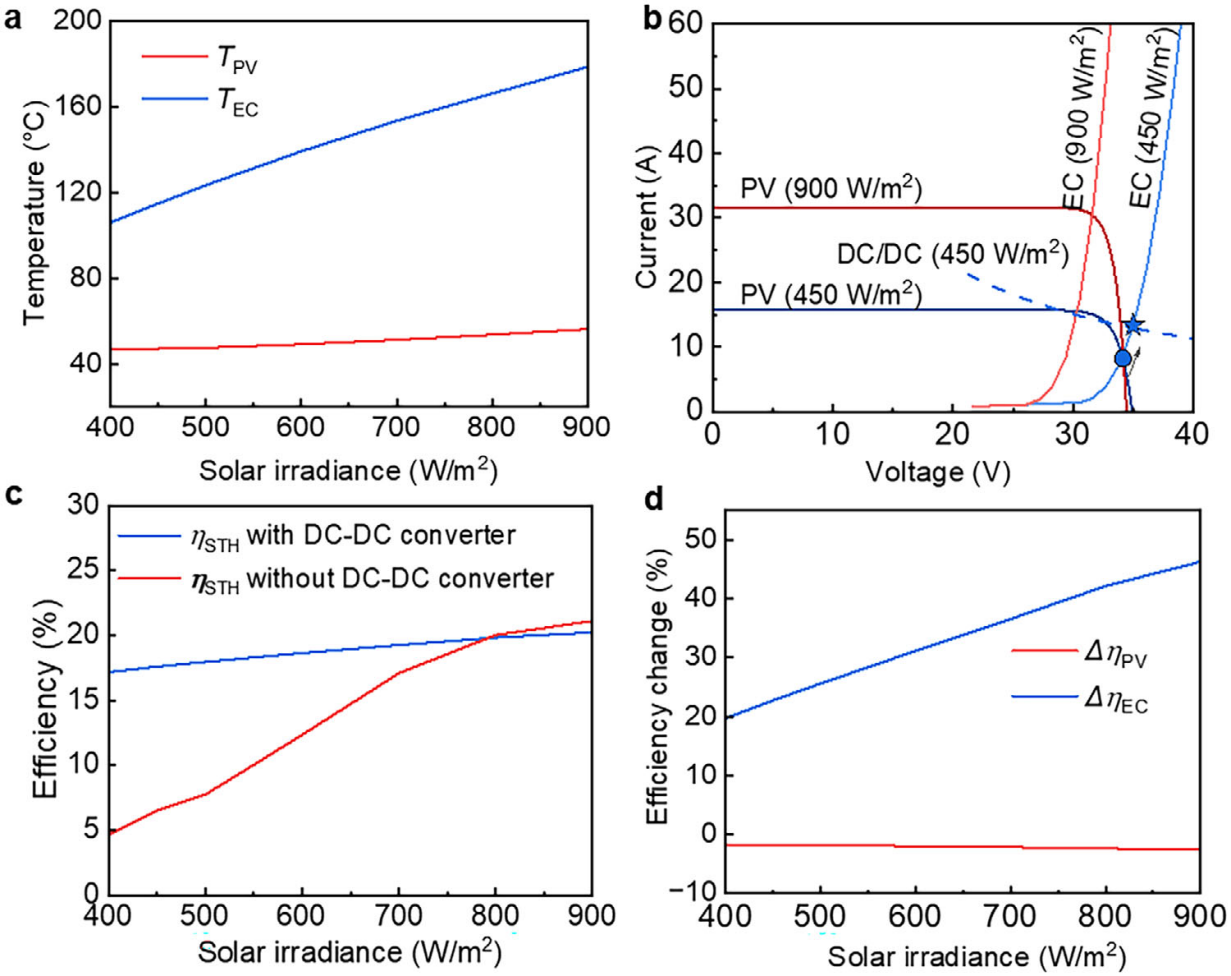
Performance of GaAs‐based SSPVTH system under different solar irradiances. a) PV and electrolyzer temperatures under different solar irradiances. Solar irradiance significantly impacts the temperature of the electrolyzer, which in turn affects its efficiency. b) Diagram of changing the operation point from the blue dot to the blue star through a DC‐DC converter. c) Comparison of STH efficiency with and without a DC‐DC converter at various solar irradiances. The DC‐DC converter can maintain the STH efficiency in a stable state. When irradiance is reduced to 450 W m^−^^2^, the STH efficiency remains at 17.6%, a significant improvement compared to the 6.5% efficiency without the DC‐DC converter. However, due to the efficiency loss of the DC‐DC converter (5%), when irradiance exceeds ≈800 W m^−^^2^, the losses incurred by the converter surpass those experienced without it. d) Efficiency changes of the PV and electrolyzer (EC) compared to the standard conditions at various solar irradiances.

The above results are based on the standard AM1.5D solar spectrum. However, in practical outdoor environments, solar irradiance changes over time, affecting the PV power output and the temperature of the modeled SSPVTH system, thereby impacting the overall system performance. Therefore, it is important to analyze the impact of solar irradiance on the system performance and find a solution to minimize the possible negative impact of various solar irradiances. **Figure** **5**a illustrates the temperature range of PV and electrolyzer of the GaAs‐based SSPVTH when the solar irradiance varies from 400 W m^−^^2^ to 900 W/m^2^. The temperature of the PV does not change significantly because it is continuously cooled by the HTF through HX1 to produce hot water. While reduced solar irradiance lowers hot water production, the PV temperature remains within a stable range. However, when the sun is low in the sky, the solar irradiance drops below 400 W m^−^^2^, approximately equivalent to air mass 6 (AM 6), and the waste heat from the PV is no longer sufficient to produce 50 °C hot water. The reduction of solar irradiance has a more pronounced impact on the electrolyzer. As solar irradiance decreases from 900 to 450 W m^−^^2^ (equivalent to AM 5), the temperature of the electrolyzer drops from 179.6 °C to around only 115 °C. This leads to two significant effects. First, the efficiency of the electrolyzer decreases. Second, and more critically, the efficiency loss due to the mismatch of I‐V curves between the PV and electrolyzer greatly increases, as detailed in Figure 5b. As the solar irradiance and electrolyzer temperature decrease, the efficiency of the electrolyzer declines, and the voltage required at the same current density increases, which is reflected in the rightward movement of the electrolyzer I‐V curve. This causes the OP to move away from the MPP, resulting in the electrolyzer not being able to fully utilize the power of the PV, forcing the operation at a lower PV power level. This leads to a decrease in the actual operating efficiency of the PV. When the solar irradiance drops to 450 W m^−^^2^, the STH efficiency decreases to only 6.5%, and the PV efficiency at the OP falls to ≈8%.

To address this issue, a DC‐DC converter is added to the electrical circuit between the PV and the electrolyzer to ensure that the power from the PV is always obtained at the MPP. The I‐V curve of the DC‐DC converter is shown as the dotted line in Figure 5b, and its efficiency is assumed to be 95% in this study.^[^ ^30^ ^]^ In this way, the electrolyzer can always receive power at the MPP of the PV, eliminating efficiency losses caused by the mismatching in a reduced solar irradiance. The position of the OP changes from the intersection of the I‐V curve of the PV and the I‐V curve of the electrolyzer (the blue dot in the figure) to the intersection of the I‐V curve of the DC‐DC converter and the I‐V curve of the electrolyzer (the blue star in the figure). After adding the DC‐DC converter, the STH efficiency at 450 W/m^2^ significantly increased from 6.5% to 17.6%, enabling the SSPVTH system to retain a relatively high STH efficiency for different solar irradiances.

The DC‐DC converter effectively addresses the mismatch problem in the SSPVTH system caused by variations in solar irradiance. As shown in Figure 5c, the DC‐DC converter ensures that the STH efficiency remains stable. However, because the DC‐DC converter has a 5% efficiency loss when the irradiance exceeds 800 W/m^2^, the converter's losses become greater than those experienced without using it. After adding the DC‐DC converter, the highest STH efficiency at 900 W m^−^^2^ is 20.2%. Which is slightly lower than the 21.1% STH efficiency without a DC‐DC converter. Figure 5d illustrates the efficiency changes of the PV cells relative to the standard condition (25 °C and AM1.5) and the efficiency changes of the electrolyzer relative to the standard condition (30 °C) under different solar irradiances. While solar irradiance has a slight impact on PV efficiency, the electrolyzer performs significantly better at higher solar irradiances.

### 2.5 The Impact of Design Parameters

The results presented above are based on selected parameters, including a 17× concentration ratio, 5% optical loss of the spectral‐splitting optical filter, and 20‐mm‐thick thermal insulation layers. To further understand system performance, it is valuable to investigate the impact of these parameters on the SSPVTH system.

**Figure 6:**
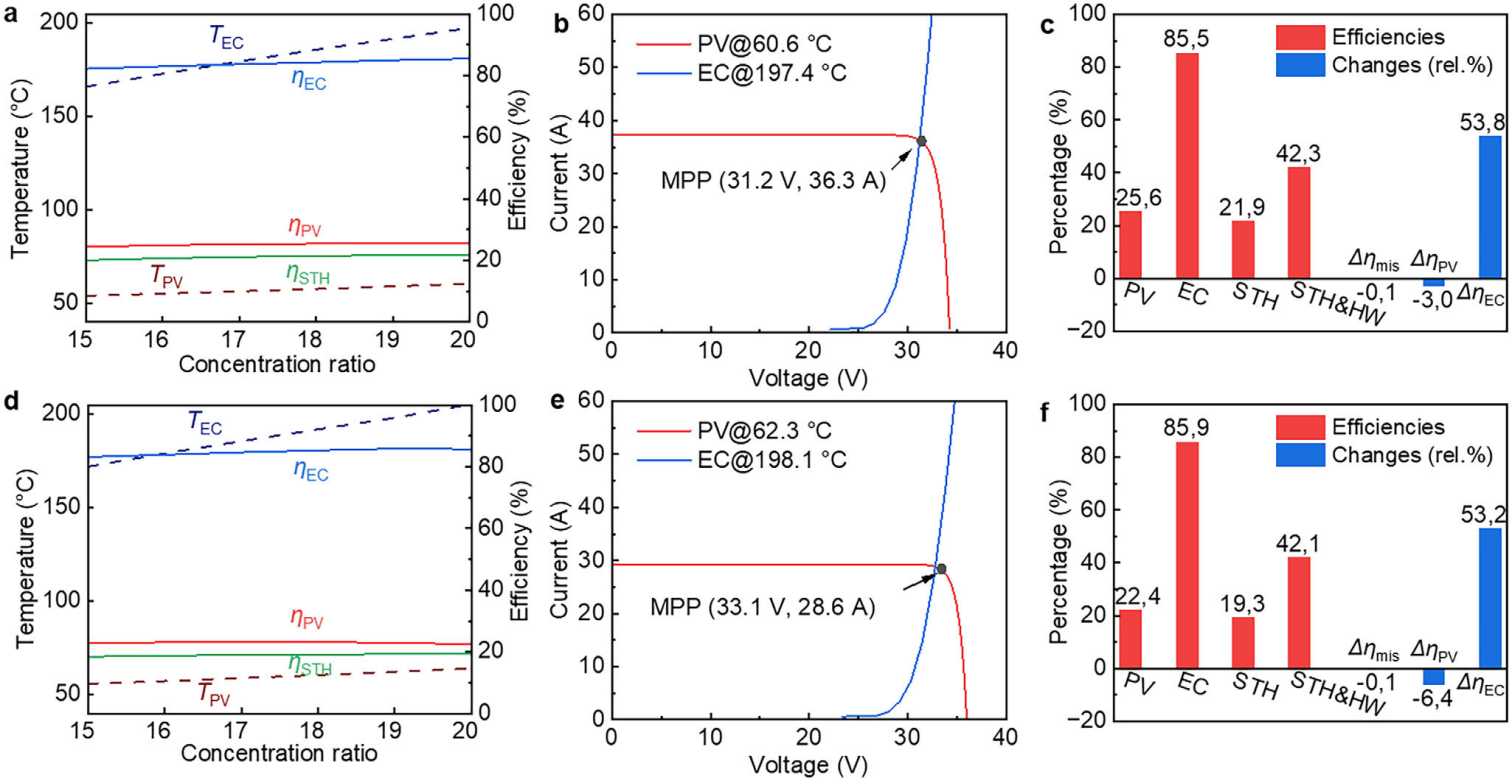
Performance of SSPVTH systems under different solar concentration ratios. a) Effect of concentration ratio on GaAs PV and electrolyzer performance. The electrolyzer temperature surpasses 200 °C when the concentration ratio exceeds 20×. b) Current‐voltage characteristics of the GaAs PV and electrolyzer at the optimal concentration (20×). c) Efficiencies of the GaAs PV, electrolyzer, STH, and solar‐to‐hydrogen & hot water (STH&HW) at the optimal concentration (20×). A maximum STH efficiency of 21.9% is achieved under this configuration. d) Effect of concentration ratio on perovskite PV and electrolyzer performance. The electrolyzer temperature surpasses 200 °C when the concentration ratio exceeds 19×. e) Current‐voltage characteristics of the perovskite PV and electrolyzer at the optimal concentration (19×). f) Efficiencies of the perovskite PV, electrolyzer, STH, and STH&HW at the optimal concentration (19×). Due to the higher temperature coefficient of perovskite PV, its efficiency decreases as the concentration ratio reaches 19×. However, the rising electrolyzer temperature enhances its efficiency, leading to a slight increase in the overall STH efficiency to 19.3%.

The concentration ratio is a critical factor in solar concentrating systems. Parabolic trough solar concentrators typically operate within a concentration ratio range of 10–30×.^[^ ^31^ ^]^ The performance of SSPVTH systems using GaAs and perovskite PVs under varying concentration ratios is shown in **Figure** **6**. As illustrated in Figure 6a, the temperature and efficiency of the GaAs PV and electrolyzer, along with the corresponding STH efficiency, are evaluated for concentration ratios ranging from 15× to 20×. Although the rise in operating temperature of GaAs cells with increasing concentration ratio, the overall impact remains beneficial, leading to enhanced electrical efficiency. Similarly, the electrolyzer temperature rises with increasing solar concentration, enhancing its efficiency. Consequently, the system's STH efficiency follows a positive trend, increasing from 20.1% at 15× to 21.9% at 20×. Although solar dish concentrators can achieve concentration ratios exceeding 100×, further increases would lead to overheating and potential damage to the electrolyzer. The membrane‐less electrolyzer used in this study is designed for temperatures up to 200 °C, which is reached when the solar concentration ratio is 20×. Therefore, the maximum concentration ratio in this study is limited to 20×. Figure 6b,c provide details on the current‐voltage characteristics, efficiencies, and efficiency losses of each component at 20×, representing the upper‐efficiency limit under the current operating conditions.

The impact of the concentration ratio on the perovskite‐based SSPVTH system is shown in Figure 6d. It should be noted that the upper solar concentration limit for this system is 19×, beyond which the electrolyzer temperature exceeds its limit 200 °C. Therefore, the system cannot work at a higher concentration ratio. At the optimal concentration ratio (19×), the perovskite PV achieves an electrical efficiency of 23.0%, slightly higher than the 22.9% at 15×. Figure 6b,c provide further insights into the I‐V characteristics, efficiencies, and efficiency losses at the optimal concentration ratio (19×). The system's STH efficiency reaches 19.3% at 19×.

**Figure 7:**
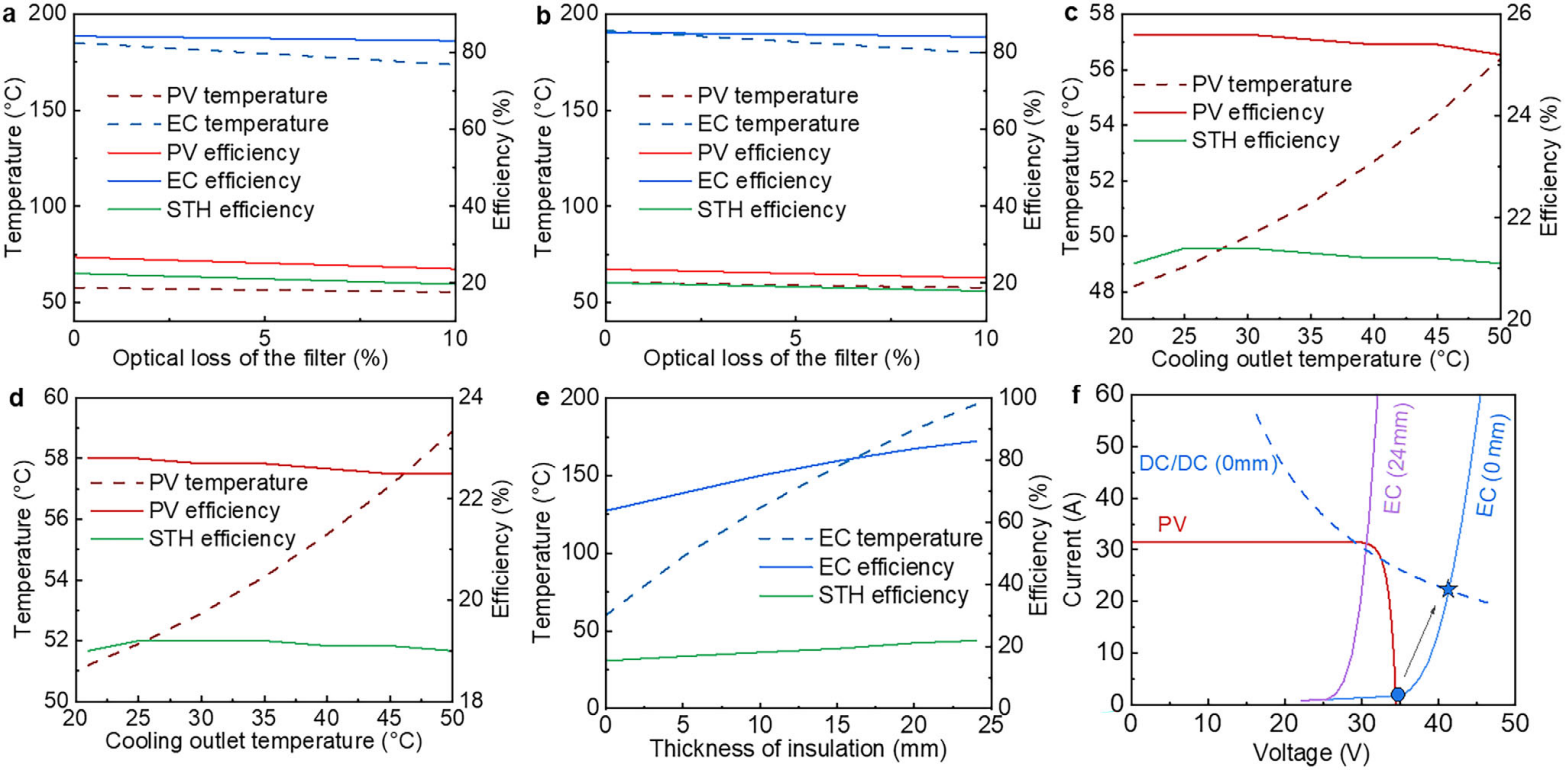
Impact of optical and thermal adjustments on system performance. a) Effect of optical losses in the spectral‐splitting filter on GaAs‐based SSPVTH system performance. b) Effect of optical losses in the spectral‐splitting filter on perovskite‐based SSPVTH system performance. c) Effect of PV cooling channel outlet temperature on GaAs‐based SSPVTH system performance. d) Effect of PV cooling channel outlet temperature on perovskite‐based SSPVTH system performance. e) Effect of thermal insulation thickness on electrolyzer performance and STH efficiency in the GaAs‐based SSPVTH system. f) Current‐voltage curves of the GaAs PV and electrolyzer for thermal insulation thicknesses of 0 and 24 mm, with a DC‐DC converter used to match the PV and electrolyzer.

**Figure** **7**a illustrates the impact of spectral‐splitting optical filter losses on the performance of the GaAs‐based SSPVTH system. In the ideal case (0% optical loss), the PV, electrolyzer, and STH efficiencies reach 26.7%, 84.3%, and 22.5%, respectively. However, as optical losses increase from 0% to 5% and 10%, the STH efficiency declines from 22.5% to 21.1% and 19.7%, emphasizing the critical importance of minimizing optical losses for maximizing overall system efficiency. Similarly, Figure 7b presents the effect of optical filter losses on the perovskite‐based SSPVTH system, which follows a comparable trend. As optical losses increase from 0% to 5% and 10%, the STH efficiency decreases from 20.1% to 19.0% and 17.9%. This further underscores the need for optimizing optical filter design to enhance system performance.

The PV efficiency can be improved by further cooling the PV cells, leading to enhanced overall system performance. In the previous sections, the outlet temperature of the PV cooling channel was set to 50 °C to ensure the system could generate heat suitable for domestic hot water applications. However, if domestic hot water generation is no longer required, the PV cells can be cooled to lower temperatures by increasing the cooling mass flow rate. Figure 7c illustrates the effect of cooling channel outlet temperature on PV temperature, PV efficiency, and STH efficiency in the GaAs‐based SSPVTH system. When the cooling mass flow rate is significantly increased, reducing the cooling outlet temperature from 50.0 to 21.0 °C, the PV temperature decreases from 56.4 °C to 48.2 °C, and the GaAs PV efficiency increases from 25.2% to 25.6%. However, due to the low‐temperature coefficient of GaAs cells, this improvement is relatively minor. Consequently, the STH efficiency remains at 21.1%. Although further cooling can marginally improve system performance, this approach has two key drawbacks: i) if the cooling channel outlet temperature drops below 50 °C, the system can no longer provide hot enough water for domestic use. This results in a loss of ≈20% of the incident solar energy that could otherwise be utilized for water heating; ii) A higher cooling flow rate requires greater pump power, which increases energy consumption. For instance, the pump power consumption increased by 2.5 times to cool the PV from 56.4 to 48.2 °C, with no additional STH efficiency improvement. Similarly, Figure 7d shows the effect of cooling channel outlet temperature on PV temperature, PV efficiency, and STH efficiency in the perovskite‐based SSPVTH system. When the cooling outlet temperature is reduced from 50.0 to 21.0 °C, the PV temperature decreases from 58.9 to 51.2 °C, and the perovskite PV efficiency increases from 22.5% to 22.8%.

Thermal losses significantly impact electrolyzer performance and, consequently, overall system efficiency. The electrolyzer temperature can be regulated by adjusting the thermal insulation thickness of the electrolyzer stack and connection pipes, as illustrated in Figure 7e. When the thermal insulation thickness is reduced from 24 to 0 mm, the electrolyzer operating temperature drops from 200 °C (its upper‐temperature limit) to 60 °C. This thermal loss directly affects the electrolyzer's current‐voltage characteristics, as shown in Figure 7f, where the current‐voltage curve shifts to the right with decreasing insulation thickness. Despite DC‐DC converter adjustments, the STH efficiency still experiences a substantial decline from 21.9% to 15.4%, primarily due to the significant reduction in electrolyzer efficiency from 86.1% to 63.7%. These results underscore the critical role of thermal management in maintaining high system performance,^[^ ^32^ ^]^ highlighting the need for adequate thermal insulation to minimize heat loss and optimize electrolyzer efficiency. Preliminary economic analysis indicates that SSPVTH systems can achieve hydrogen production costs comparable to Si‐PVH, particularly with high‐efficiency, long‐lifetime GaAs PVs.^[^ ^33^ ^]^ The concentrating design enhances the STH efficiency while reducing PV area requirements, ensuring competitiveness even with costly PV materials. Further improvements in PV durability and manufacturing efficiency could enhance SSPVTH's viability for large‐scale hydrogen production.

## 3 Conclusion

An SSPVTH system is designed and modeled, which combines concentrated PV, solar thermal, spectral‐splitting filter, and a membrane‐less electrolyzer stack to produce green hydrogen. This design achieves spectral splitting by using different wavelengths for PV power generation and an evacuated tube absorber (ETA) to generate solar thermal. Electricity and high‐temperature solar thermal are then simultaneously used for electrolyzer hydrogen production. Using GaAs PV, the system achieves a solar‐to‐hydrogen (STH) efficiency of 21.1%. Compared to a conventional PV hydrogen (PVH) system, the SSPVTH system demonstrates a relatively 31.1% improvement in STH efficiency. In the SSPVTH system using perovskite PV, the STH efficiency reaches 19.0%. Under different solar irradiances, the performance of the SSPVTH system varies significantly, and the STH efficiency drops notably at low irradiance levels. To address this issue, a DC‐DC converter was added to the system. This addition allows the system to achieve an STH efficiency of 17.6% even at half the standard irradiance (450 W m^−^^2^), a substantial improvement over the 6.5% STH efficiency without the DC‐DC converter. This enhancement enables the SSPVTH system to maintain high performance under varying solar irradiances, aligning with real weather conditions and allowing the system to efficiently produce hydrogen in a real‐world environment. Incorporating both GaAs and perovskite PVs highlights the SSPVTH system's flexibility and compatibility with both current high‐performance and emerging PV technologies. Silicon solar cells can also be a good candidate for the SSPVTH system in future research, especially under relatively low solar concentration ratios.

Despite its promising performance, the SSPVTH system has areas requiring further technical development in future work. Membrane‐less electrolyzers, while suitable for high‐temperature operation, experience challenges such as ohmic losses and gas crossover. Future work should focus on optimizing electrolyte flow, electrode materials, and system designs to address these issues to further enhance the STH efficiency. It can also be interesting to incorporate energy storage technologies, such as phase‐change materials or batteries, to store excess thermal or electrical energy for consistent operation during periods of low solar irradiance. Additionally, field testing under real‐world conditions is critical for validating system performance and addressing deployment challenges. Direct experimental validation of the complete SSPVTH system remains challenging due to the lack of available data for this novel design. Further experimental investigations are essential to generate reliable datasets that can comprehensively validate the system model and provide empirical support. Additionally, the current model incorporates several assumptions—for instance, the series resistance loss between solar cells is neglected.

Overall, the SSPVTH system represents a significant advancement in solar hydrogen production by synergizing advanced spectral and thermal management strategies with emerging electrolyzer technologies. It achieves substantial efficiency improvements over conventional methods, demonstrating a robust design adaptable to various photovoltaic technologies and operating conditions.

## 4 Experimental Section

The energy conversion process of the solar hydrogen systems in this study is simulated using in‐house codes based on energy balance and thermodynamics principles. The governing equations for various models, including optical, thermal, electrical, and electrochemical calculations, are detailed in the Supporting Information.

### Optical and Thermal Modeling

The light distribution model calculates solar energy absorption in different system components. Since concentrated PV systems only utilize direct sunlight, the AM1.5D spectrum (900 W m^−2^) is applied. The thermal model accounts for heat transfer between the glazing glass, PV module, ETA, and membrane‐less electrolyzer.^[^ ^34^, ^35^, ^36^ ^]^ The heat exchange processes are modeled using the logarithmic mean temperature difference (LMTD) method,^[^ ^37^ ^]^ ensuring accurate predictions of PV cooling performance and electrolyte heating efficiency. System heat losses due to convection, radiation, and conduction are also incorporated.^[^ ^36^, ^38^ ^]^

### Electrical and Electrochemical Modeling

The PV module performance is simulated using a single‐diode model, incorporating the calculation of dark saturation current^[^ ^34^ ^]^ based on bandgap energy and temperature effects, the spectral response function for light‐generated current determination,^[^ ^26^ ^]^ and temperature‐dependent voltage loss, which considers the fill factor (*FF*) and temperature coefficients for open‐circuit voltage (*V*_oc_) and short‐circuits current (*I*_sc_).^[^ ^12^, ^39^ ^]^ The membrane‐less electrolyzer stack is modeled using an electrochemical framework, where the total cell voltage accounts for the thermodynamic potential (*E*_₀_) determined by Gibbs free energy,^[^ ^24^ ^]^ ohmic losses influenced by electrolyte conductivity and electrode spacing, and overpotentials for the hydrogen evolution reaction (HER) and oxygen evolution reaction (OER), which are computed using the Tafel equation.^[^ ^40^, ^41^ ^]^ The PV and electrolyzer models are iteratively solved until convergence is reached, ensuring that the current voltage (I‐V) characteristics of the PV module and electrolyzer stack are well‐matched.

### Efficiency Parameters Definition

The solar‐to‐hydrogen efficiency (*η*_STH_) quantifies the conversion of incident solar energy into the chemical energy of hydrogen, defined as the ratio of hydrogen output power (*P*_H2_) to the total incident solar energy on the system (*P*_in_).^[^ ^13^, ^42^ ^]^

(1) $$\eta_{\mathrm{STH}}=\frac{P_{H2}}{P_{\mathrm{in}}}=\frac{\frac{I_{\mathrm{op}}\eta_{F}}{nF}N_{S\_\mathrm{EC}}\Delta H_{H2}}{P_{\mathrm{in}}}\;$$

where *I*_op_ is the current of the electrolyzer at the operating point, *F* is the Faraday constant, *n* is the number of exchanged electrons in the reaction, *η*_F_ is the Faraday efficiency, *N*_S_EC_ is the number of electrolyzer cells connected in series. To further analyze performance losses, three efficiency deviation parameters are introduced: the mismatch efficiency loss (*Δη*_mis_), which represents the relative efficiency loss due to the deviation of the electrolyzer operating point from the PV's maximum power point (MPP):

(2) $$\Delta \;\eta_{\mathrm{mis}}=\frac{\eta_{\mathrm{PV}\;}-\;\eta_{\mathrm{PV},\mathrm{MPP}}}{\eta_{\mathrm{PV},\mathrm{MPP}}}\;$$

where *η*_PV,MPP_ is the efficiency of PV at MPP under working temperature. The PV temperature degradation loss (*Δη*_T_PV_), which accounts for the reduction in PV efficiency as a result of temperature increases compared to standard test conditions (25 °C), *η*_PV,MPP@std_:

(3) $$\Delta \;\eta_{T\_\mathrm{PV}}=\frac{\eta_{\mathrm{PV},\mathrm{MPP}}-\;\eta_{\mathrm{PV},\mathrm{MPP}@\mathrm{std}}}{\eta_{\mathrm{PV},\mathrm{MPP}@\mathrm{std}}}\;$$

and the electrolyzer temperature gain (*Δη*_T_EC_), which quantifies the relative improvement in electrolyzer efficiency when operating at elevated temperatures, *η*_EC_, compared to its performance at 30 °C, *η*_EC@std_:

(4) $$\Delta \;\eta_{T\_\mathrm{EC}}=\frac{\eta_{\mathrm{EC}\;}-\;\eta_{\mathrm{EC}@\mathrm{std}}}{\eta_{\mathrm{EC}@\mathrm{std}}}\;$$

These parameters collectively characterize the interactions between optical, thermal, and electrochemical processes, providing insight into the impact of system integration strategies on overall hydrogen production efficiency. This integrated modeling framework enables a comprehensive assessment of SSPVTH system performance across different operating conditions, with further details on governing equations and parameter values available in the Supporting Information.

## Conflict of Interest

The authors declare no conflict of interest

## Author Contributions

Y.T., P.H., G.H., and B.S.R. developed the concept and research methodology. Y.T. simulates and analyzes the performance of the system. P.H. simulated the performance of the electrolyzer. Y.T., N.A., P.H., and G.H. analyze data. Y.T., G.H., and B.S.R contributed to writing and revising the manuscript. G.H. and B.S.R. directed the project.

## Supporting information



Supporting Information

## Data Availability

The data that support the findings of this study are available from the corresponding author upon reasonable request.

## References

[1] Z. Li , S. Fang , H. Sun , R. J. Chung , X. Fang , J. H. He , Adv. Energy Mater. 2023, 13, 2203019.

[2] T. T. Le , P. Sharma , B. J. Bora , V. D. Tran , T. H. Truong , H. C. Le , P. Q. P. Nguyen , Int. J. Hydrogen Energy 2024, 54, 791.

[3] C. Gunathilake , I. Soliman , D. Panthi , P. Tandler , O. Fatani , N. A. Ghulamullah , D. Marasinghe , M. Farhath , T. Madhujith , K. Conrad , Y. Du , M. Jaroniec , Chem. Soc. Rev. 2024, 53, 10900. 39421882 10.1039/d3cs00731f

[4] W. Noor , M. D. Amin , Future Energy 2024, 3, 1.

[5] International Energy Agency (IEA) , Global Hydrogen Review 2023, https://www.iea.org/reports/global‐hydrogen‐review‐2023, (accessed: June 2024).

[6] International Renewable Energy Agency (IRENA), https://www.irena.org/Energy‐Transition/Technology/Hydrogen, (accessed: June 2024).

[7] A. Godula‐Jopek , Hydrogen Production: By Electrolysis, John Wiley & Sons, Hoboken, NJ, USA 2015.

[8] National Renewable Energy Laboratory (NREL), https://www.nrel.gov/pv/cell‐efficiency.html, (accessed: June 2024).

[9] K. Branker , M. J. M. Pathak , J. M. Pearce , Renewable Sustainable Energy Rev. 2011, 15, 4470.

[10] J. Kim , H. Lee , S. Park , Y. Jeong , Energies 2021, 14, 4278.

[11] E. J. Nordberg , M. J. Caley , L. Schwarzkopf , Sol. Energy 2021, 228, 586.

[12] M. Herrando , K. Wang , G. Huang , T. Otanicar , O. B. Mousa , R. A. Agathokleous , C. N. Markides , Prog. Energy Combust. Sci. 2023, 97, 101072.

[13] I. Holmes‐Gentle , S. Tembhurne , C. Suter , S. Haussener , Nat. Energy 2023, 8, 586.

[14] A. Fallisch , L. Schellhase , J. Fresko , M. Zedda , J. Ohlmann , M. Steiner , A. Bösch , L. Zielke , S. Thiele , F. Dimroth , T. Smolinka , Int. J. Hydrogen Energy 2017, 42, 26804.

[15] A. Nakamura , Y. Ota , K. Koike , Y. Hidaka , K. Nishioka , M. Sugiyama , K. Fujii , Appl. Phys. Express 2015, 8, 107101.

[16] W. Li , H. Tian , L. Ma , Y. Wang , X. Liu , X. Gao , Mater. Adv. 2022, 3, 5598.

[17] S. Senthilraja , R. Gangadevi , R. Marimuthu , M. Baskaran , Int. J. Hydrogen Energy 2020, 45, 7498.

[18] E. Akrami , A. Nemati , H. Nami , F. Ranjbar , Int. J. Hydrogen Energy 2018, 43, 622.

[19] A. Hauch , S. D. Ebbesen , S. H. Jensen , M. Mogensen , J. Mater. Chem. 2008, 18, 2331.

[20] H. Liang , F. Wang , Z. Chen , S. Yong , B. Lin , Y. Pan , Sol. Energy 2020, 206, 84.

[21] H. Liang , F. Wang , Z. Dong , D. Zhang , Z. Cheng , C. Zhang , B. Lin , H. Xu , 2020, Energy 194, 116913.

[22] H. Liang , R. Su , W. Huang , Z. Cheng , F. Wang , G. Huang , D. Yang , Energy Convers. Manage. 2022, 252, 115049.

[23] S. M. H. Hashemi , P. Karnakov , P. Hadikhani , E. Chinello , S. Litvinov , C. Moser , D. Psaltis , Energy Environ. Sci. 2019, 12, 1592.

[24] P. Hadikhani , S. M. H. Hashemi , S. A. Schenk , D. Psaltis , Sustain. Energy Fuels 2021, 5, 2419. 33997295 10.1039/d1se00255d PMC8095110

[25] M. A. Green , E. D. Dunlop , M. Yoshita , N. Kopidakis , K. Bothe , G. Siefer , X. Hao , Prog. Photovolt. 2023, 31, 7.

[26] Y. Chaibi , M. Salhi , A. El‐Jouni , A. Essadki , Sol. Energy 2018, 163, 376.

[27] Y. Chaibi , A. Allouhi , M. Malvoni , M. Salhi , R. Saadani , Sol. Energy 2019, 188, 1102.

[28] T. J. Silverman , M. G. Deceglie , B. Marion , S. Cowley , B. Kayes , S. Kurtz , in Proc. 2013 IEEE 39th Photovoltaic Specialists Conf. (PVSC), IEEE, 2013, pp. 0103–0108.

[29] Y. Zhao , F. Ma , Z. Qu , S. Yu , T. Shen , H. X. Deng , J. You , Science 2022, 377, 531. 35901131 10.1126/science.abp8873

[30] M. Takagi , K. Shimizu , T. Zaitsu , in Proc. APEC. Seventeenth Annual IEEE Applied Power Electronics Conf. and Exposition (Cat. No. 02CH37335), Vol. 2, IEEE, 2002, pp. 735–741.

[31] A. Fernández‐García , E. Zarza , L. Valenzuela , M. Pérez , Renew. Sustain. Energy Rev. 2010, 14, 1695.

[32] G. Huang , K. Wang , C. N. Markides , Light Sci. Appl. 2021, 10, 28. 33542174 10.1038/s41377-021-00465-1 PMC7862645

[33] A. Grimm , W. A. de Jong , G. J. Kramer , Int. J. Hydrogen Energy 2020, 45, 22545.

[34] J. Peacock , G. Huang , J. Song , C. N. Markides , Energy Convers. Manage. 2022, 269, 116071.

[35] M. Herrando , C. N. Markides , K. Hellgardt , Appl. Energy 2014, 122, 288.

[36] G. Notton , C. Cristofari , M. Mattei , P. Poggi , Appl. Therm. Eng. 2005, 25, 2854.

[37] X. Cui , K. J. Chua , M. R. Islam , W. M. Yang , Energy Convers. Manage. 2014, 88, 372.

[38] I. Guarracino , A. Mellor , N. J. Ekins‐Daukes , C. N. Markides , Appl. Therm. Eng. 2016, 101, 778.

[39] P. Dobreva , E. E. van Dyk , F. J. Vorster , Sol. Energy 2021, 227, 116.

[40] M. Grdeń , G. Jerkiewicz , Electrocatalysis 2019, 10, 173.

[41] D. Chanda , J. Hnát , A. S. Dobrota , I. A. Pašti , M. Paidar , K. Bouzek , Phys. Chem. Chem. Phys. 2015, 17, 26864. 26399740 10.1039/c5cp04238k

[42] R. H. Coridan , A. C. Nielander , S. A. Francis , M. T. McDowell , V. Dix , S. M. Chatman , N. S. Lewis , Energy Environ. Sci. 2015, 8, 2886.

