# Supplementary material for: Hybrid Solar Spectral‐Splitting Photovoltaic‐Thermal Hydrogen Production Systems
Source: Adv Sci (Weinh). 2025 Apr 27;12(28):2503205. doi: 10.1002/advs.202503205 (PMC12302634; doi:10.1002/advs.202503205)
Supplement: Supplementary file 1 — Supporting Information [file ADVS-12-2503205-s001.pdf]

ADVANCED SCIENCE Open Access

Supporting Information

for *Adv. Sci.*, DOI 10.1002/advs.202503205

Hybrid Solar Spectral-Splitting Photovoltaic-Thermal Hydrogen Production Systems

Yu Tian, Pooria Hadikhani, Nada Alati, Bryce S. Richards* and Gan Huang**

Supporting Information

Hybrid Solar Spectral-Splitting Photovoltaic-Thermal Hydrogen Production Systems

Yu Tian, Pooria Hadikhani, Nada Alati, Bryce S. Richards*, Gan Huang**

1. Mathematical calculation methods

The energy conversion process of the solar hydrogen systems in this study is simulated using in-house codes based on principles of energy balance and thermodynamics. The governing equations for various models are detailed below.

1.1. Light distribution model

For concentrated PV designs, only direct sunlight can be concentrated, while diffuse light is disregarded. Consequently, the terrestrial air mass 1.5 direct (AM1.5 D) spectrum at an intensity of 900 W/m2 is utilized in this study. The solar irradiance of $S_{AM1.5D}$ can be calculated by,

$$S_{\mathrm{AM1.5D}} = \int_{280\ \mathrm{nm}}^{4000\ \mathrm{nm}} G_{\mathrm{AM1.5D}}\,(\lambda)\mathrm{d}\lambda \tag{1}$$

where $G_{AM1.5D}$ is the spectral irradiance of AM1.5D. The light initially concentrated through the parabolic reflector. Subsequently, the light spectrum is divided into two components by the spectrum-splitting optical filter: the solar irradiance that penetrates the filter and reaches PV, S_{PV}, and the solar irradiance that is reflected by the filter and reaches ETA, S_{ETA}:

$$S_{\mathrm{PV}} = \rho_{\mathrm{PR}} \frac{A_{\mathrm{PR}}}{A_{\mathrm{PV}}} \int_{280\ \mathrm{nm}}^{4000\ \mathrm{nm}} \tau_{\mathrm{F}}(\lambda) G_{\mathrm{AM1.5D}}(\lambda)\mathrm{d}\lambda \tag{2}$$

$$S_{\mathrm{ETA}} = \rho_{\mathrm{PR}} \frac{A_{\mathrm{PR}}}{A_{\mathrm{ST}}} \int_{280\ \mathrm{nm}}^{4000\ \mathrm{nm}} \rho_{\mathrm{F}}(\lambda) I_{\mathrm{AM1.5D}}(\lambda)\mathrm{d}\lambda \tag{3}$$

where τ_F the transmittance of the filter, ρ_F the reflection of the filter, A_{PR} the area of the parabolic reflector, A_{PV} the area of the PV, A_{ST} the area of the inner solar thermal absorber of the ETA, ρ_{PR} is the reflection of the parabolic reflector. The ratio of the area of the parabolic reflector and the area of ST inside ETA determines the geometric concentration ratio for ETA. Due to the difference in the surface area between the outer glass and the inner solar thermal absorber of the ETA, the solar irradiance experienced by the outer glass, $S_{ETA,og}$,

$$S_{\mathrm{ETA,og}} = \rho_{\mathrm{PR}} \frac{A_{\mathrm{PR}}}{A_{\mathrm{og}}} \int_{280\ \mathrm{nm}}^{4000\ \mathrm{nm}} \rho_{\mathrm{F}}(\lambda) G_{\mathrm{AM1.5D}}(\lambda)\mathrm{d}\lambda \tag{4}$$

where A_{og} is the area of the outer glass of the ETA. Assuming that the spectrum-splitting optical filter splits light ideally and its absorption loss is 5%. Then the values of τ_F and ρ_F as the function of wavelength are:

$$\tau_F(\lambda) = \begin{cases} 0.95 & \lambda < \lambda_{cut} \\ 0 & \lambda > \lambda_{cut} \end{cases} \tag{5}$$

$$\rho_F(\lambda) = \begin{cases} 0 & \lambda < \lambda_{cut} \\ 0.95 & \lambda > \lambda_{cut} \end{cases} \tag{6}$$

where λ_{cut} is the cutting edge of the spectrum-splitting filter (λ_{cut} = 900 nm in this study).

1.2. Electrical model of PV

Firstly, the dark saturation current, J_{ds}, is calculated as[7] :

$$J_{ds} = k_1 T_{std}^{\frac{3}{z}} \exp\left(\frac{-E_g}{b k_B T_{std}}\right) \tag{7}$$

where k_1, b and z are empirical parameters for PV (k_1 = 0.03, b = 1.2, and z = 0.98)[7] , E_g is the band gap energy for PV material, k_B the Stefan-Boltzmann constant, and T_{std} is the standard temperature of PV (*i.e.*, 298.15 K). The light generated current, J_L, is determined by integrating the spectral response of the PV cells as:

$$J_L = \rho_{PR} \frac{A_{PR}}{A_{PV}} \tau_g \alpha_{PV} A_{cell} \int_{280}^{4000} \tau_F(\lambda) G(\lambda) SR(\lambda) d\lambda \tag{8}$$

where $SR(\lambda)$ is the spectral response of PV, $G(\lambda)$ is the incident spectrum, τ_g is the transmittance of the glass covering the PV and α_{PV} is absorption of PV. All the optical losses of the parabolic reflector, spectral-splitting filter, cover glass of PV and solar cell surface are considered. A_{cell} is the area of one PV cell in PV module. The short-circuit current, J_{sc}, can be calculated by:

$$J_{SC} = A_j J_L \tag{9}$$

where A_j is the ideality factor for short-circuit current (A_j = 0.97 in this study) [8] . The relationship between the voltage, V, and current of each cell, J, can be derived from the ideal diode equation:

$$J = J_{sc} - J_{ds} \exp\left[\frac{eV}{A' k_B T_{std}}\right] \tag{10}$$

where A' is the PV cell ideality factor (A'=1 in this study)[7] , e is the charge of an electron. The I-V curve of the PV module is derived by interconnecting multiple PV cells in series and/or parallel configurations. Assuming negligible wire losses in this study (series resistance loss was assumed to be ignored), the performance parameter translation from a single cell to the entire module is considered to be unity:

$$I_{PV} = J N_{p\_PV} \tag{11}$$

$$V_{PV} = V N_{s\_PV} \tag{12}$$

where $N_{\text{p\_PV}}$ and $N_{\text{s\_PV}}$ are the numbers of PV cells connected in parallel and series ($N_{\text{P\_PV}}$ = 2 and $N_{\text{S\_PV}}$ = 30 in this study). The influence of temperature on PV performance primarily manifests in three aspects: current, voltage, and fill factor (*FF*). Notably, the effect of temperature elevation on voltage surpasses its impact on current[8], [18]. The open-circuit voltage (V_{oc}) at various temperatures can be determined using the following formula:

$$V_{\text{oc}} = V_{\text{oc\_std}}\left(1 + \beta_{\text{V}}(T_{\text{PV}} - T_{\text{std}})\right) \tag{13}$$

where $V_{\text{oc\_std}}$ is the open circuit voltage at standard temperature, and β_{v} is the temperature coefficient of voltage (β_{v} = - 0.14 %/°C in this study[19]), T_{PV} is the temperature of PV panel under the working condition, and T_{std} is the standard temperature, in this study, it is 25 °C. The effect of temperature on short circuit current (I_{sc}) and FF can be calculated in the same way:

$$I_{\text{SC}} = I_{\text{sc\_std}}\left(1 + \beta_{\text{I}}(T_{\text{PV}} - T_{\text{std}})\right) \tag{14}$$

$$FF = FF_{\text{std}}\left(1 + \beta_{\text{FF}}(T_{\text{PV}} - T_{\text{std}})\right) \tag{15}$$

where $I_{\text{sc\_std}}$ and F_{std} are the short circuit current and fill factor at standard temperature. β_{I} and β_{FF} are the temperature coefficients of current and fill factor (β_{I} = 0.07%/°C and β_{FF} = -0.01%/°C in this study[19]). The final effect of temperature on efficiency can be seen as the sum of the effects of temperature on the three[1]:

$$\beta_{\text{Eff}} = \beta_{\text{FF}} + \beta_{\text{I}} + \beta_{\text{V}} \tag{16}$$

After determining the short-circuit current and open-circuit voltage, another important point to determine is the maximum power point (MPP), which can be calculated by the fill factor:

$$FF = \frac{V_{\text{MPP}} I_{\text{MPP}}}{V_{\text{oc}} I_{\text{SC}}} \tag{17}$$

Since the effect of temperature on voltage is much greater than its effect on current[8], [18], the current at MPP can be expressed by Anderson translation equation[9]:

$$I_{\text{MPP}} = I_{\text{MPP\_std}}\left(1 + \beta_{\text{I}}(T_{\text{PV}} - T_{\text{std}})\right) \tag{18}$$

where $I_{\text{MPP\_std}}$ is the MPP current at standard temperature. By substituting the above equations for V_{oc}, I_{sc}, and I_{MPP} into the equation for *FF*, the voltage value of MPP at different temperatures, V_{MPP} then can be calculated:

$$V_{\text{MPP}} = V_{\text{MPP\_std}}\left(1 + \beta_{\text{FF}}(T_{\text{PV}} - T_{\text{std}})\right)\left(1 + \beta_{\text{V}}(T_{\text{PV}} - T_{\text{std}})\right) \tag{19}$$

The circuit losses in the connections between solar cells in the solar module are negligible due to the emerging back-surface connection technology, which utilizes very compact connectors to link the solar cells.

1.3. Electrochemical model of the membrane-less electrolyzer

In this model, the catalyst used for the electrolysis reaction is pure nickel. The thickness of these nickel sheets is 0.3 mm. The flat nickel sheets are cut into an area of 5 cm², the size of the

electrodes, and inserted into the electrolyzer. Afterwards, the catalyst undergoes multiple cyclic voltammetry (where the potential is increased linearly from an initial potential to a final potential and then back to the initial potential) for activation. The ratio of the electrode area in the electrolysis cell to the light absorption area of PV cells [3] is 0.4875 for the SSPVTH system with GaAs PV and 0.4354 for the SSPVTH system with perovskite PV. The pressure of electrolyzer at each temperature is the minimum value that prevents the boiling of the electrolyte. An electrochemical model of electrolysis can be used to describe the I-V relationship of an electrolyzer. The voltage required for the electrolysis reaction consists of the following components[6] [3] :

$$V = E_0 + \eta'_{\mathrm{ohmic}} + \eta'_{\mathrm{HER}} + \eta'_{\mathrm{OER}} + \eta'_{\mathrm{con}} + \eta'_{\mathrm{bub}} \tag{20}$$

Among them, E_0 is the starting potential, which is the minimum potential required for the electrolysis reaction to occur; η'_{ohmic} is the ohmic losses overpotential, which is the overpotential required to overcome the resistance between electrodes filled with liquid electrolyte; η'_{HER} and η'_{OER} are hydrogen evolution reaction and oxygen evolution reaction overpotentials. η'_{con} is the overpotential due to the dissolved gas concentration, and η'_{bub} is the overpotential due to bubble evolution. The influence of concentration and bubble overpotentials on the electrochemical reaction is small at the high electrolyte flow rates considered in this study. Therefore, η'_{con} and η'_{bub} are not considered in the calculations:

$$V = E_0 + \eta'_{\mathrm{ohmic}} + \eta'_{\mathrm{HER}} + \eta'_{\mathrm{OER}} \tag{21}$$

The starting potential is a function of the Gibbs free energy change:

$$E_0 = -\frac{\Delta G}{nF} \tag{22}$$

where ΔG is the change in Gibbs free energy in the water splitting reaction; n is the number of exchanged electrons in the reaction (n = 2 for water splitting); F is Faraday's constant. The value of E_0 is 1.23 V under standard conditions (298.15 Kelvin, 1 atmosphere)[6] .The values of E_0 at different temperatures required for calculation are based on data from previous studies[10] . Ohmic losses are calculated as follows:

$$\eta'_{\mathrm{ohmic}} = \frac{jW}{\sigma'} \tag{23}$$

where j is the current density; W is the interelectrode distance, which is set as 2.5 mm in this study; σ' is the conductivity of the electrolyte. The electrolyte is KOH at concentration of 30 % (w/w) in water solution in this study, with σ' = 610 mS·cm^{-1} at 30 °C. Values of σ' at different temperatures refer to previous research[11] . The activation overpotentials of HER and OER are calculated by Tafel equation respectively[12] , [13] :

$$\eta'_{\mathrm{HER}} = \beta_{\mathrm{HER}} \log\left(\frac{j}{j_{0\_\mathrm{HER}}}\right) \tag{24}$$

$$\eta'_{\mathrm{OER}} = \beta_{\mathrm{OER}} \log\left(\frac{j}{j_{0\_\mathrm{OER}}}\right) \tag{25}$$

where j_{0_HER} and j_{0_OER} are the respective exchange current density; β_{HER} and β_{OER} are the Tafel slopes for each reaction. The values of j_{0_HER}, j_{0_OER}, β_{HER} and β_{OER} for different temperature can be found in the literature[12] -[14] .

In the above calculations, the free energy change (ΔG), conductivity of the KOH solution (σ), exchange current densities (j_{0_HER}, j_{0_OER}) and Tafel slopes (β_{HER}, β_{OER}) are all functions of the temperature. Therefore, the j-V curves at different temperatures can then be calculated from the parameters at respective temperatures.

After the above calculation, the j-V curve can be obtained. For the I-V relationship of each cell, the current can be calculated using the area of the electrode:

$$I_{\mathrm{EC\_cell}} = jA_{\mathrm{ele}} \tag{26}$$

where A_{ele} is the electrode area (5 cm^2 for each electrolyzer cell in this study). For each electrolyzer stack, the overall I-V curve depends on the number of cells connected in series ($N_{\mathrm{s\_EC}}$, 18 for GaAs and 19 for perovskite) in each unit, and the number of units connected in parallel ($N_{\mathrm{p\_EC}}$, 13 for GaAs and 11 for perovskite) in the stack, as described before. The value of $N_{\mathrm{s\_EC}}$ depends on the I-V curve of the PV under ideal conditions, so that the OP is as close to the MPP as possible. The value of $N_{\mathrm{p\_EC}}$ depends on the target current density of the electrolyzer. The overall current and voltage calculations are as follows:

$$I_{\mathrm{EC\_stack}} = I_{\mathrm{EC\_cell}} N_{\mathrm{p\_EC}} \tag{27}$$

$$V_{\mathrm{EC\_stack}} = V_{\mathrm{EC\_cell}} N_{\mathrm{s\_EC}} \tag{28}$$

In common electrolyzers, the membrane is the main component that degrades at high temperatures. In this study, removing the membrane in membrane-less electrolyzers allows operation at temperatures as high as 180 °C. This temperature falls within the intermediate range for electrolyzers, which typically operate between 100 and 200 °C. The electrode and casing materials of the membrane-less electrolyzer must withstand this temperature range and be compatible with a 30% KOH solution. The electrode material of the membrane-less electrolyzer is nickel, which can operate under these conditions. Additionally, the casing material is made of stainless steel, with sealing components made of Teflon, both of which are suitable for operation at intermediate temperatures with a 30% KOH solution.

1.4. Thermal model of the system

The thermal model is based on the law of energy balance for each component. Detailed equations for the glazing glass surrounding the PV module, the PV module, are introduced below.

The energy input of the glazing glass is the absorbed solar irradiance $Q_{in,g}$, which comes from incident sunlight and reflection light from the PV module. The energy output includes radiative and convective heat transfer to the ambient, $Q_{r,g-sky}$ and $Q_{conv,g-a}$, respectively, and conductive and radiative heat transfer to the PV module, $Q_{cd,g-PV}$ and $Q_{r,g-PV}$. The energy balance equation for the glazing glass surrounding the PV module is expressed as,

$$Q_{\mathrm{in,g}} = Q_{\mathrm{r,g-sky}} + Q_{\mathrm{conv,g-a}} + Q_{\mathrm{cd,g-PV}} + Q_{\mathrm{r,g-PV}} \tag{29}$$

corresponding to,

$$\begin{aligned} A_{\mathrm{PV}}\alpha_{\mathrm{g}}S_{\mathrm{PV}}\left(1+\tau_{\mathrm{g}}\rho_{\mathrm{PV}}\right) &= \varepsilon_{\mathrm{g}}\sigma A_{\mathrm{PV}}\left(T_{\mathrm{g}}^{4}-T_{\mathrm{sky}}^{4}\right) \\ &+h_{\mathrm{wind}}A_{\mathrm{PV}}\left(T_{\mathrm{g}}-T_{\mathrm{a}}\right)+h_{\mathrm{cd,g-PV}}A_{\mathrm{PV}}\left(T_{\mathrm{g}}-T_{\mathrm{PV}}\right)+\frac{A_{\mathrm{PV}}\sigma\left(T_{\mathrm{g}}^{4}-T_{\mathrm{PV}}^{4}\right)}{\frac{1}{\varepsilon_{\mathrm{PV}}}+\frac{1}{\varepsilon_{\mathrm{g}}}-1} \end{aligned} \tag{30}$$

where S_{PV} is the solar irradiance, A_{PV} is the surface area of PV module (the same as the area of the glazing glass), α_g is the absorptivity of glazing glass (α_g = 0.03[7]), τ_g is the transmissivity of the glazing glass (0.95[7]), and ρ_{PV} is the reflectivity of PV module (0.07[7]), σ is the Stefan–Boltzmann constant (1.38 ×10^{-23} m^2 kg s^{-2} K^{-1}), ε_g is the emissivity of glass (0.9[7]), and ε_{pv} is the emissivity of PV module (0.9[20]). T_g, T_{PV}, T_a and T_{sky} are cover glass, PV, ambient and sky temperatures respectively. T_{sky} is expressed as T_{sky} = $0.0552T_a$[21] .The h_{wind} is the heat convection coefficient to the ambient, which is a function of wind speed[15] . $h_{cd,g\text{-}PV}$ is the heat transfer coefficient between the glazing glass and the PV module, dominating by the heat conduction of the air gap between the glazing glass ang the PV module[15] . The energy balance for PV module follows:

$$Q_{\mathrm{in,PV}} + Q_{\mathrm{cd,g-PV}} + Q_{\mathrm{r,g-PV}} = Q_{\mathrm{ele-PV}} + Q_{\mathrm{conv,PV-HTF}} \tag{31}$$

corresponding to,

$$\begin{aligned} A_{\mathrm{PV}}\alpha_{\mathrm{pv}}\tau_{\mathrm{g}}S_{\mathrm{PV}}+h_{\mathrm{cd,g-PV}}A_{\mathrm{PV}}\left(T_{\mathrm{g}}-T_{\mathrm{PV}}\right)+\frac{A_{\mathrm{PV}}\sigma\left(T_{\mathrm{g}}^{4}-T_{\mathrm{PV}}^{4}\right)}{\frac{1}{\varepsilon_{\mathrm{PV}}}+\frac{1}{\varepsilon_{\mathrm{g}}}-1} \\ =\eta_{\mathrm{ele}}\tau_{\mathrm{g}}G_{\mathrm{PV}}A_{\mathrm{PV}}+h_{\mathrm{conv,PV-HTF}}A_{\mathrm{pv}}\left(T_{\mathrm{PV}}-T_{\mathrm{ave,HTF}}\right) \end{aligned} \tag{32}$$

where η_{ele} is the electrical efficiency of PV module, $h_{conv,PV\text{-}HTF}$ is the heat transfer coefficient between the PV module and the HTF in the cooling channel[7] . The average temperature of the HTF in the cooling channel is calculated by:

$$T_{\mathrm{ave,HTF}} = \frac{T_{\mathrm{out,PV}} + T_{\mathrm{in,PV}}}{2} \tag{33}$$

where $T_{\mathrm{out,PV}}$ and $T_{\mathrm{in,PV}}$ are the outlet and inlet temperature of HTF in the cooling channel. The energy balance for the HTF in the cooling channel is:

$$Q_{\mathrm{conv,PV-HTF}} = Q_{\mathrm{gain,HTF}} + Q_{\mathrm{loss,PV-a}} \tag{34}$$

corresponding to,

$$\begin{aligned} & h_{\mathrm{conv,PV-HTF}} A_{\mathrm{PV}} \left(T_{\mathrm{PV}} - T_{\mathrm{ave,HTF}}\right) \\ = \ & \dot{m}_{\mathrm{HTF}} c_{\mathrm{HTF}} \left(T_{\mathrm{out,PV}} - T_{\mathrm{in,PV}}\right) + h_{\mathrm{loss,HTF-a}} A_{\mathrm{PV}} \left(T_{\mathrm{ave,HTF}} - T_{\mathrm{a}}\right) \end{aligned} \tag{35}$$

where $h_{\mathrm{conv,PV\text{-}HTF}}$ is the heat transfer coefficient between the PV module and the HTF in the cooling channel, $\dot{m}_{\mathrm{HTF}}$ is the mass flow rate, c_{HTF} is the specific heat capacity of the HTF (water as HTF for PV cooling, 4186 J/(kg·K) at around 50 °C[22]), $h_{\mathrm{loss,HTF\text{-}a}}$ is the equivalent heat transfer coefficient between the HTF and the ambient. Additionally, all heat losses incurred during fluid flow through the connection piping are accounted for by employing the subsequent energy balance equation to determine the temperature of the outflow:

$$\dot{m}_i c_i \left(T_{\mathrm{out,i}} - T_{\mathrm{in,i}}\right) = h_{\mathrm{loss,pipe-a}} A_{\mathrm{p}} \left(\frac{T_{\mathrm{out,i}} + T_{\mathrm{in,i}}}{2} - T_{\mathrm{a}}\right) \tag{36}$$

where $\dot{m}_i$ and c_i are the mass flow rate and specific heat capacity of the fluid in a pipe, for cooling water, c_{water} is 4186 J/(kg·K), for HTF in the ETA at around 180°C, c_{HTF} is 2663 J/(kg·K)[23] . $T_{\mathrm{out,i}}$ and $T_{\mathrm{in,i}}$ are the outlet and inlet of a connection pipe. A_{P} is the surface area of a pipe (set as 1 m^2 in this study). $h_{\mathrm{loss,pipe\text{-}a}}$ is the equivalent heat transfer coefficient between the HTF and the ambient.

Given the absence of thermal coupling between PV and the ETA, the waste heat generated by the PV system is exclusively utilized for 50 °C hot water production, without contributing to electrolyte heating. The heat exchange 1 (HX1) is used to transfer the PV heat for domestic hot water generation (heat the water from 20 °C to 50 °C). The common logarithmic average of the temperature difference (LMTD) method is used to model the heat exchange process of HX1.[24] .The energy balance of outer glass of the ETA includes absorbed solar irradiance, Q_{og}, the radiative heat from the inner solar thermal absorber, $Q_{\mathrm{r,og\text{-}ST}}$, radiative heat loss, $Q_{\mathrm{r,og\text{-}sky}}$, and convective heat loss, $Q_{\mathrm{conv,og\text{-}sky}}$ to the environment:

$$Q_{\mathrm{og}} = Q_{\mathrm{r,og-sky}} + Q_{\mathrm{conv,og-a}} + Q_{\mathrm{r,og-ST}} \tag{37}$$

corresponding to,

$$\begin{aligned} & A_{\mathrm{og}} \alpha_{\mathrm{g}} S_{\mathrm{ETA,og}} \left(1 + \tau_{\mathrm{g}} \rho_{\mathrm{ST}}\right) = \varepsilon_{\mathrm{g}} \sigma A_{\mathrm{og}} \left(T_{\mathrm{g}}^4 - T_{\mathrm{a}}^4\right) \\ & + h_{\mathrm{wind}} A_{\mathrm{og}} \left(T_{\mathrm{g}} - T_{\mathrm{a}}\right) + \frac{A_{\mathrm{og}} \sigma \left(T_{\mathrm{g}}^4 - T_{\mathrm{ST}}^4\right)}{\frac{1}{\varepsilon_{\mathrm{ST}}} + \frac{1}{\varepsilon_{\mathrm{g}}} - 1} \end{aligned} \tag{38}$$

where ρ_{ST} (0.08) and ε_{ST} (0.08) are the reflectivity and emissivity of the solar thermal absorber[7]. T_{ST} is the average temperature of solar thermal absorber. The energy balance of solar thermal absorber consists of absorbed solar irradiance, Q_{ST}, radiative heat to outer glass, $Q_{r,og\text{-}ST}$, and conductively heat to the HTF, $Q_{conv,ST\text{-}HTF}$,

$$Q_{\mathrm{ST}} + Q_{\mathrm{r,og-ST}} = Q_{\mathrm{conv,ST-HTF}} \tag{39}$$

corresponding to,

$$A_{\mathrm{ST}}\alpha_{\mathrm{ST}}\tau_{\mathrm{g}}S_{\mathrm{ETA}} + \frac{A_{\mathrm{ST}}\sigma\left(T_{\mathrm{g}}^{4} - T_{\mathrm{ST}}^{4}\right)}{\frac{1}{\varepsilon_{\mathrm{ST}}} + \frac{1}{\varepsilon_{\mathrm{g}}} - 1} = h_{\mathrm{conv,ST-HTF}}A_{\mathrm{ST}}\left(T_{\mathrm{ST}} - T_{\mathrm{ave,ETA}}\right) \tag{40}$$

where $T_{ave,EAT}$ is the average temperature of inlet, $T_{in,ETA}$, and outlet temperature, $T_{out,ETA}$, of HTF through ST channel,

$$T_{\mathrm{ave,ETA}} = \frac{T_{\mathrm{out,ETA}} + T_{\mathrm{in,ETA}}}{2} \tag{41}$$

The $h_{conv,ST\text{-}HTF}$ is the heat transfer coefficient for $Q_{conv,ST\text{-}HTF}$. The convective heat transfer coefficient can be estimated based on the Nusselt number:

$$h_{\mathrm{conv,ST-HTF}} = \frac{Nu \times k_{\mathrm{HTF}}}{D_{\mathrm{ST}}} \tag{42}$$

where the Nu is 3.66 the cylindrical pipes used in ETA with laminar flow [7], [15]. The D_{ST} is the diameter of the inner tube of the ETA (2.8cm), and k_{HTF} is thermal conductivity of the HTF (0.12 W·(m/K)[23]). Upon establishing the energy balance of the ETA, the Heat HTF is conveyed via pipes to the heat exchanger 2 (HX2), where it is utilized to heat the electrolyte. The LMTD method is used to model the heat exchange process of HX2[24]. Thermal energy input inside the electrolyzer is only generated through the overpotential during electrolysis, $Q_{heat,EC}$. which is used to heat the electrolyte (KOH solution), $Q_{conv,EC\text{-}KOH}$ as well as heat loss to the environment, $Q_{conv,EC\text{-}a}$. The energy balance in the electrolyzer is expressed as,

$$Q_{\mathrm{heat,EC}} = Q_{\mathrm{conv,EC-KOH}} + Q_{\mathrm{conv,EC-a}} \tag{43}$$

corresponding to,

$$I_{\mathrm{wp}}\left(V_{\mathrm{wp}} - V_{\mathrm{TN}}N_{\mathrm{S\_EC}}\right) = \dot{m}_{\mathrm{EC}}c_{EC}\left(T_{\mathrm{out,EC}} - T_{\mathrm{in,EC}}\right) + A_{\mathrm{EC}}h_{\mathrm{loss,EC-a}}\left(T_{\mathrm{ave,EC}} - T_{\mathrm{a}}\right) \tag{44}$$

$$T_{\mathrm{ave,EC}} = \frac{T_{\mathrm{in,EC}} + T_{\mathrm{out,EC}}}{2} \tag{45}$$

where I_{wp} is the working current of electrolyzer stack, V_{wp} is the working voltage of electrolyzer stack, and V_{TN} is the thermal neutral voltage of electrolytic water reaction. It exhibits minimal variation with temperature and is considered as a constant in this study, set at 1.48V.[16] $\dot{m}_{EC}$

is the flow rate of the electrolyte, c_{EC} is the specific heat capacity of electrolyte, set as 3.28 kJ/(kg·K), $T_{in,EC}$ is the temperature of the electrolyte at the electrolyzer inlet, $T_{out,EC}$ is the temperature of the electrolyte at the electrolyzer outlet. The temperature of the entire electrolyzer stack is considered as uniformly distributed, and its temperature, T_{ave_EC}, is the average temperature of the electrolyte inside the electrolyzer. A_{EC} is the surface area of the entire electrolyzer stack. The $h_{loss,\ EC-a}$ is the equivalent heat transfer coefficient for $Q_{conv,EC-a}$, calculated as,

$$h_{\mathrm{loss,ec-a}} = \frac{1}{\frac{W_{\mathrm{shell}}}{k_{\mathrm{shell}}} + \frac{W_{\mathrm{insu,EC}}}{k_{\mathrm{insu,EC}}} + \frac{1}{h_{\mathrm{wind}}}} \tag{46}$$

where W_{shell} (2mm) and $W_{insu,EC}$ (2 cm) are the thickness of shell layer and insulation layer of electrolyzer, and k_{shell} (30 W·(m/K)[27]) and $k_{insu,EC}$ (0.035 W·(m/K)[28]) are the thermal conductivity of them.

Additional water needs to be added into the electrolyte to make up for the water consumed in the hydrogen production process. After adding additional water, the temperature is calculated as:

$$T_{\mathrm{out,EC\_mix}} = \frac{\dot{m}_{\mathrm{loss}}}{\dot{m}_{\mathrm{EC}}} T_{\mathrm{in,EC\_mix}} + \frac{\dot{m}_{\mathrm{EC}} - \dot{m}_{\mathrm{loss}}}{\dot{m}_{\mathrm{EC}}} T_{\mathrm{out,EC}} \tag{47}$$

where T_{out,EC_mix} is the electrolyte temperature after mixing additional water, T_{in,EC_mix} is the temperature of additional cold water, set as 20 °C in this study. $\dot{m}_{EC}$ is the flow rate of consumed water in the electrolyzer (the same as the additional water).

1.5. Efficiency parameters definition

Based on Faraday's laws of electrolysis, it is possible to calculate the rate of hydrogen production on a molar basis[2] ,

$$\dot{n} = \frac{I_{\mathrm{op}}\eta_{\mathrm{F}}}{nF} N_{\mathrm{S\_EC}} \tag{48}$$

where I_{op} is the current of electrolyzer at operating point, F is the Faraday constant, n is the number of exchanged electrons in the reaction, in electrolysis water reaction is 2[2] . The η_F is the Faraday efficiency. Typically, the membrane-less electrolyzer exhibits high Faradaic efficiency; therefore, here it is assumed to be 1. The power of the output fuel is defined as,

$$P_{\mathrm{H2}} = \dot{n}\Delta H_{\mathrm{H2}} \tag{49}$$

where ΔH_{H2} is the enthalpy of reaction, equals to 286 kJmol^{-1}. The computations for hydrogen power output and hydrogen production efficiency in this research are grounded on the enthalpy of reaction. The solar-to-hydrogen efficiency, η_{STH} is defined as:[17]

$$\eta_{\mathrm{STH}} = \frac{P_{\mathrm{H2}}}{P_{\mathrm{in}}} = \eta_{\mathrm{PV}}\,\eta_{\mathrm{EC}} \tag{50}$$

where P_{in} is the total energy from the solar irradiance, consists of the solar energy used for hydrogen generation, $P_{sun,H2}$, and for auxiliary system, $P_{sun,aux}$, i.e., energy consumption for pumps and solar trackers.

$$P_{\mathrm{in}} = P_{\mathrm{sun,H2}} + P_{\mathrm{sun,aux}} \tag{51}$$

$$P_{\mathrm{sun,H2}} = G_{\mathrm{AM1.5D}} A_{\mathrm{PR}} \tag{52}$$

The auxiliary system is energized by a standby independent PV system, with its total power requirement calculated as the sum of the power consumption across all components,

$$P_{\mathrm{sun,aux}} = \frac{P_{\mathrm{aux}}}{\eta_{\mathrm{PV-aux}}} \tag{53}$$

where $\eta_{PV\text{-}aux}$ is the efficiency of standby independent PV system, assumed to be 20% in this study. The value of P_{aux} is 22.85 W (1.09 W for a solar tracker[25], 21.76 W for four pumps[26]). Solar to hydrogen efficiency can be divided into two parts: PV efficiency at operating point, η_{PV}, and electrolyzer efficiency at operating point, η_{EC},

$$\eta_{\mathrm{PV}} = \frac{P_{\mathrm{ele}}}{P_{\mathrm{in}}} = \frac{I_{\mathrm{op}} V_{\mathrm{op}}}{P_{\mathrm{sun,H2}} + P_{\mathrm{sun,aux}}} \tag{54}$$

$$\eta_{\mathrm{EC}} = \frac{P_{\mathrm{H2}}}{P_{\mathrm{ele}}} = \frac{\dot{n}\Delta H_{\mathrm{H2}}}{I_{\mathrm{op}} V_{\mathrm{op}}} \tag{55}$$

where P_{ele} is the electricity power output of PV at operating point.

After specifying the efficiency definitions, to facilitate a more detailed analysis of how various factors influence the STH efficiency under working conditions, several additional parameters are introduced. The $\Delta\eta_{mis}$ quantifies the PV efficiency variation resulting from the operational point's deviation from the MPP:

$$\Delta\eta_{\mathrm{mis}} = \frac{\eta_{\mathrm{PV}} - \eta_{\mathrm{PV,MPP}}}{\eta_{\mathrm{PV,MPP}}} \tag{56}$$

where $\eta_{PV,MPP}$ is the efficiency of PV at MPP under working temperature. The second additional parameter measures the variation in PV efficiency attributable to temperature fluctuations at the maximum power point, $\eta_{PV,MPP}$, relative to the efficiency variation observed at the MPP under standard temperature conditions (25 °C), $\eta_{PV,MPP@std}$,

$$\Delta\eta_{\mathrm{T\_PV}} = \frac{\eta_{\mathrm{PV,MPP}} - \eta_{\mathrm{PV,MPP@std}}}{\eta_{\mathrm{PV,MPP@std}}} \tag{57}$$

The third addtioanl parameter assesses the variation in electrolyzer efficiency due to shifts in the actual operating temperature of the electrolyzer, in comparison to its efficiency at standard temperature, $\eta_{EC@std}$, which set as 30 °C in this study,

$$\Delta\eta_{\mathrm{T\_EC}} = \frac{\eta_{\mathrm{EC}} - \eta_{\mathrm{EC@std}}}{\eta_{\mathrm{EC@std}}} \tag{58}$$

Given that the efficiency of the electrolyzer is influenced by both the current density and the flow rate of the electrolyte, the standard efficiency ($\eta_{\mathrm{EC@std}}$) is consistently derived under identical conditions of current density and flow rate as those applied to η_{EC}. Therefore, $\Delta\eta_{\mathrm{T\_EC}}$ only reflects the impact of temperature variations on the efficiency of the electrolyzer system.

2. Preliminary techno-economic analysis of PVH and SSPVTH systems

The discounted cash flow approach accounts for the time value of money by applying a discount factor (DF_t) to all costs and hydrogen production over the system's lifetime:[34]

$$DF_t = \frac{1}{(1+r)^r} \tag{59}$$

$$\text{Totla costs} = I_0 + \sum_{t=1}^{n} \frac{OM_t}{(1+r)^t} + \sum_{t=1}^{n} \frac{F_t}{(1+r)^t} \tag{60}$$

Where I_0 is Initial capital costs, including the cost of photovoltaic panels, reflectors, spectral-splitting filters, electrolyzers, and other equipment. OM_t is annual operating and maintenance costs at year t, expressed as a fraction of the total capital cost. F_t is replacement costs at year t, particularly for components such as the electrolyzer that require replacement during the system's lifetime. H_t is annual hydrogen production at year t, determined by STH efficiency and system parameters. r is discount rate, which accounts for the time value of money. n is system lifetime in years.

The annual hydrogen production H_t is calculated based on the solar irradiance incident on the system and the STH efficiency:

$$H_t = \frac{I \cdot A \cdot \eta_{STH}}{HHV} \tag{61}$$

Where I is annual solar irradiance (kWh/m^2 /year). A is the effective area of the reflector (for SSPVTH) or photovoltaic panels (for PVH) (m^2). HHV the higher heating value of hydrogen as 39.4 kWh/kg. Total hydrogen production is calculated as:

$$\text{Totla hydrogen production} = \sum_{t=1}^{n} \frac{H_t}{(1+r)^t} \tag{62}$$

The levelized cost of hydrogen (LCOH) is calculated using a discounted cash flow approach. The methodology considers the total system capital costs (CAPEX), annual operating and maintenance costs (OPEX), the system's hydrogen production over its lifetime, and the time value of money through a discount rate. The formula expresses as:[34]

$$LCOH = \frac{I_0 + \sum_{t=1}^{n} \frac{OM_t + F_t}{(1+r)^t}}{\sum_{t=1}^{n} \frac{H_t}{(1+r)^t}} \quad (63)$$

The system costs are estimated based on literature reports and commercial product data, as summarized in Table S1. All assumptions are made conservatively to ensure a realistic and practical evaluation.

Table S1: Summery of costs for SSPVTH and PVH systems

Component	Unit	Price	Reference
GaAs PV	$/W	4	[29]
Perovskite PV	$/W	0.5	[30]
Si PV	$/W	0.4	[31]
Parabolic reflector	$/m^2	100	[7]
ETA	$/m^2	100	[7]
Electrolyzer	$/W	0.7	[31]
Spectral-splitting filter	$/m^2	200	[7]
Heat exchanger	$/unit	200	[7]
Pump	$/unit	100	[7]
Heat transfer fluid	$/kg	4	[32]
DC-DC converter	$/kW	50	[33]

The economic feasibility of SSPVTH systems is important for their large-scale practical deployment. We conducted a preliminary economic analysis to evaluate the levelized cost of hydrogen (LCOH) as the primary indicator of cost-effectiveness for the two SSPVTH systems and the PVH system. The LCOH represents the total cost of producing one kilogram of hydrogen over the system's lifetime, encompassing both capital expenditures (CAPEX) and operational expenditures (OPEX)[34] . The preliminary economic analysis highlights that SSPVTH systems offer significant cost advantages over non-concentrated PVH configurations when using GaAs cells. The LCOH for the GaAs-based SSPVTH system is around 7.1 $/kg H_2, representing a substantial reduction from 22.3 $/kg H_2 for the non-concentrated GaAs PVH system. This cost reduction is primarily attributed to the efficiency advantages of the SSPVTH system's concentrating design, which significantly reduces the area required for expensive GaAs PV cells while enhancing hydrogen production. It is important to note that GaAs panels are unlikely to be deployed in non-concentrated configurations due to their high cost. When

GaAs is replaced with commercial Si panels in the PVH system, the LCOH decreases to 6.3 \$/kg H_2, aligning closely with the LCOH values reported for PV-electrolyzer systems in the literature[31] [29] . Nevertheless, even with GaAs panels, the SSPVTH system achieves a competitive LCOH. For the perovskite SSPVTH system, while its PV cost is lower than that of GaAs, its LCOH is 9.0 \$/kg H_2. This is partially due to its lower STH efficiency compared to GaAs and the limited lifetime of perovskite PV panels. However, if the lifetime of perovskite PV panels could be extended to 20 years, comparable to silicon PV, its LCOH could also drop below 7.0 \$/kg H_2. These findings underscore the potential of SSPVTH systems to achieve cost competitiveness, particularly when the selected PV materials align well with the system configuration. Future advancements in GaAs manufacturing processes and cost reductions could further enhance its viability in concentrated solar applications, while improved durability of perovskite PV holds promise for further cost reductions in SSPVTH systems.

Supplementary figures

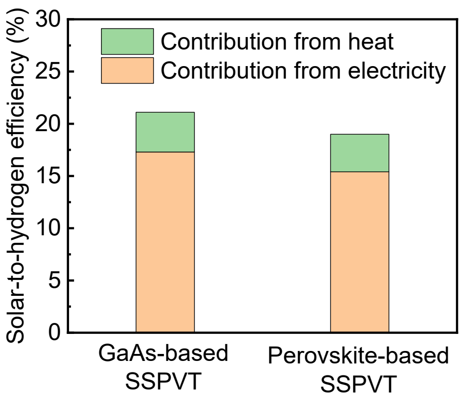

Figure S1. **Electrical and thermal contribution ratios in GaAs- and perovskite-based SSPVTH systems.** The solar-to-hydrogen (STH) efficiency is decomposed into contributions from electricity (r_{ele}) and heat (r_{heat}). For hydrogen generation from the GaAs SSPVTH system, the contribution from electricity is 81.9%, while the contribution from heat is 18.1%. For hydrogen generation from the perovskite-based SSPVTH system, the contribution from electricity is 81.1%, while the contribution from heat is 18.9%.

The values of r_{ele} and r_{heat} are calculated from the below method:

To evaluate the contribution from electricity, we define the percentage of hydrogen generation attributed to electricity as r_{ele}. This is calculated using the following equation:

$$r_{\text{ele}} = \frac{-\dot{n}\Delta G_{\text{H2@}T}}{P_{\text{in}}\eta_{\text{STH}}}$$

Where $\Delta G_{H2@T}$ is the Gibbs free energy of water electrolysis at working temperature T, and $\dot{n}$ is the mole production rate of hydrogen, P_{in} is the total incident solar energy on the system, and η_{STH} is the solar-to-hydrogen efficiency. The contribution from heat, denoted as r_{heat}, is determined by the remaining portion of the total efficiency not directly attributed to electricity, calculated as:

$$r_{\text{heat}} = 1 - r_{\text{ele}}$$

For the hydrogen generated from the GaAs SSPVTH system, the contribution of electricity is 81.9%, while the contribution from heat is 18.1%. In the case of the perovskite-based SSPVTH system, the contribution of electricity is 81.1%, and the contribution from heat is 18.9%.

References

[1] M. Herrando, K. Wang, G. Huang, T. Otanicar, O. B. Mousa, R. A. Agathokleous, C. N. Markides, *Prog. Energy Combust. Sci.* 2023, 97, 101072.

[2] I. Holmes-Gentle, S. Tembhurne, C. Suter, S. Haussener, *Nat. Energy* 2023, 8, 586.

[3] M. S. Bashir, M. A. Nadeem, M. Al-Oufi, M. Al-Hakami, T. T. Isimjan, H. Idriss, *ACS Omega* 2020, 5, 10510.

[4] C. A. Rodriguez, M. A. Modestino, D. Psaltis, C. Moser, *Energy Environ. Sci.* 2014, 7, 3828.

[5] M. A. Khan, I. Al-Shankiti, A. Ziani, H. Idriss, *Sustainable Energy Fuels* 2021, 5, 1085.

[6] P. Hadikhani, S. M. H. Hashemi, S. A. Schenk, D. Psaltis, *Sustainable Energy Fuels* 2021, 5, 2419.

[7] J. Peacock, G. Huang, J. Song, C. N. Markides, *Energy Convers. Manage.* 2022, 269, 116071.

[8] C. B. Honsberg, S. G. Bowden, Photovoltaics Education Website, www.pveducation.org, accessed: June, 2024

[9] P. Dobreva, E. E. van Dyk, F. J. Vorster, *Sol. Energy* 2021, 227, 116.

[10] F. P. Lohmann-Richters, S. Renz, W. Lehnert, M. Müller, M. Carmo, *J. Electrochem. Soc.* 2021, 168, 114501.

[11] F. Allebrod, C. Chatzichristodoulou, P. L. Mollerup, M. B. Mogensen, *Int. J. Hydrogen Energy* 2012, 37, 16505.

[12] M. Grdeń, G. Jerkiewicz, *Electrocatalysis* 2019, 10, 173.

[13] D. Chanda, J. Hnát, A. S. Dobrota, I. A. Pašti, M. Paidar, K. Bouzek, *Phys. Chem. Chem. Phys.* 2015, 17, 26864.

[14] M. H. Miles, G. Kissel, P. W. T. Lu, S. Srinivasan, *J. Electrochem. Soc.* 1976, 123, 332.

[15] M. Herrando, C. N. Markides, K. Hellgardt, *Appl. Energy* 2014, 122, 288.

[16] R. L. LeRoy, C. T. Bowen, D. J. LeRoy, *J. Electrochem. Soc.* 1980, 127, 1954.

[17] R. H. Coridan, A. C. Nielander, S. A. Francis, M. T. McDowell, V. Dix, S. M. Chatman, N. S. Lewis, *Energy Environ. Sci.* 2015, 8, 2886.

[18] Y. Chaibi, A. Allouhi, M. Malvoni, M. Salhi, R. Saadani, *Sol. Energy* 2019, 188, 1102.

[19] T. J. Silverman, M. G. Deceglie, B. Marion, S. Cowley, B. Kayes, S. Kurtz, in Proc. *2013 IEEE 39th Photovoltaic Specialists Conf. (PVSC),* IEEE, 2013, pp. 0103–0108.

[20] I. Guarracino, A. Mellor, N. J. Ekins-Daukes, C. N. Markides, *Appl. Therm. Eng.* 2016, 101, 778.

[21] G. Notton, C. Cristofari, M. Mattei, P. Poggi, *Appl. Therm. Eng.* 2005, 25, 2854.

[22] Engineering ToolBox, Water – Specific Heat Capacity, https://www.engineeringtoolbox.com/specific-heat-capacity-water-d_660.html, accessed: September, 2024.

[23] Chevron, Chevron Lubricants: Delo 400 LE SAE 15W-40, https://cglapps.chevron.com/msdspds/PDSDetailPage.aspx?docDataId=77158, accessed: August, 2024.

[24] X. Cui, K. J. Chua, M. R. Islam, W. M. Yang, *Energy Convers. Manage.* 2014, 88, 372.

[25] S. Ahmad, S. Shafie, M. Z. A. Ab Kadir, in Proc. *2012 IEEE Int. Conf. on Power and Energy (PECon),* IEEE, 2012, pp. 366–371.

[26] Wolseley, Grundfos UPS3 Circulating Pump, https://www.wolseley.co.uk, accessed: June, 2024.

[27] Engineering ToolBox, Metals, Metallic Elements and Alloys – Thermal Conductivities, https://www.engineeringtoolbox.com/thermal-conductivity-metals-d_858.html, accessed: August, 2024.

[28] Z. Pásztory, *J. Build. Eng.* 2021, 44, 102604.

[29] B. Lee, D. Fan, S. R. Forrest, *Sustainable Energy Fuels* 2020, 4, 2035.

[30] M. Cai, Y. Wu, H. Chen, X. Yang, Y. Qiang, L. Han, *Adv. Sci.* 2017, 4, 1600269.

[31] E. Vartiainen, C. Breyer, D. Moser, E. Román Medina, C. Busto, G. Masson, A. Jäger-Waldau, *Sol. RRL* 2022, 6, 2100487.

[32] Seair Exim Solutions, Therminol VP1 Import Data, https://www.seair.co.in/therminol-vp1-import-data.aspx, accessed: February, 2025.

[33] R. Sharma, H. Gao, *IEEE Trans. Power Electron.* 2006, 21, 587–591.

[34] A. Grimm, W. A. de Jong, G. J. Kramer, *Int. J. Hydrogen Energy* 2020, 45, 22545.
